# Engineering a Highly Efficient Carboligase for Synthetic One-Carbon Metabolism

**DOI:** 10.1021/acscatal.1c01237

**Published:** 2021-04-20

**Authors:** Maren Nattermann, Simon Burgener, Pascal Pfister, Alexander Chou, Luca Schulz, Seung Hwan Lee, Nicole Paczia, Jan Zarzycki, Ramon Gonzalez, Tobias J. Erb

**Affiliations:** † Department of Biochemistry & Synthetic Metabolism, Max-Planck-Institute for Terrestrial Microbiology, Karl-von-Frisch-Str. 10, 35043 Marburg, Germany; ‡ Department of Chemical and Biomedical Engineering, University of South Florida, Tampa, Florida 33620, United States; § Center for Synthetic Microbiology (SYNMIKRO), 35043 Marburg, Germany

**Keywords:** carbon fixation, one-carbon building blocks, C−C bond formation, biocatalysis, directed evolution, acyloin condensation, thiamine diphosphate, formyl-CoA

## Abstract

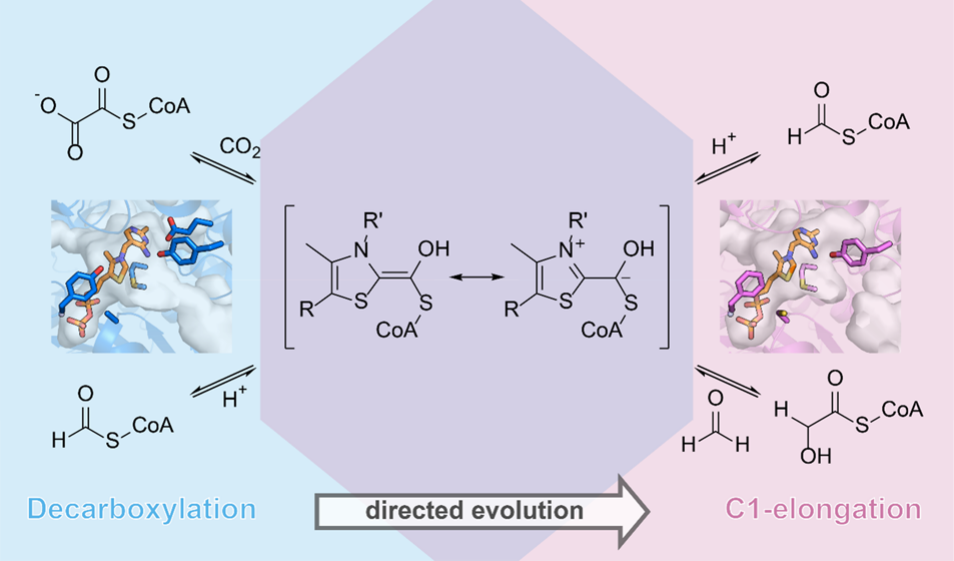

One of the biggest challenges to realize a circular carbon economy is the synthesis of complex carbon compounds from one-carbon (C1) building blocks. Since the natural solution space of C1–C1 condensations is limited to highly complex enzymes, the development of more simple and robust biocatalysts may facilitate the engineering of C1 assimilation routes. Thiamine diphosphate-dependent enzymes harbor great potential for this task, due to their ability to create C–C bonds. Here, we employed structure-guided iterative saturation mutagenesis to convert oxalyl-CoA decarboxylase (OXC) from *Methylobacterium extorquens* into a glycolyl-CoA synthase (GCS) that allows for the direct condensation of the two C1 units formyl-CoA and formaldehyde. A quadruple variant MeOXC4 showed a 100 000-fold switch between OXC and GCS activities, a 200-fold increase in the GCS activity compared to the wild type, and formaldehyde affinity that is comparable to natural formaldehyde-converting enzymes. Notably, MeOCX4 outcompetes all other natural and engineered enzymes for C1–C1 condensations by more than 40-fold in catalytic efficiency and is highly soluble in *Escherichia coli*. In addition to the increased GCS activity, MeOXC4 showed up to 300-fold higher activity than the wild type toward a broad range of carbonyl acceptor substrates. When applied in vivo, MeOXC4 enables the production of glycolate from formaldehyde, overcoming the current bottleneck of C1–C1 condensation in whole-cell bioconversions and paving the way toward synthetic C1 assimilation routes in vivo.

## Introduction

The synthesis of complex molecules from one-carbon (C1) compounds is key to a circular economy. C1 compounds, in particular formate and methanol, can be derived directly from CO_2_ through several processes, including hydrogenation, photochemistry, electrochemistry, and biocatalysis,^1−5^ and further serve as feedstock for the formation of value-added products through microbial fermentation.^6−9^ Because natural methylo- and formatotrophic microorganisms are not well suited for large-scale biotechnological processes, current efforts aim at engineering well-established microbial platform organisms by implementing natural and new-to-nature C1 converting pathways into these microbes.^10−13^

As highlighted by several recent studies, linear C1 converting pathways are particularly interesting^10,12,14^ since they minimally interfere with the host metabolism and, unlike cyclic pathways, are robust toward the draining of intermediates. However, linear pathways require the direct condensation of two C1 units, which is chemically challenging, due to the difficulty to generate C1 nucleophiles (in contrast to C1 electrophiles, which are readily available, notably in the form of CO_2_ and formaldehyde). Only two enzymes are known that catalyze direct C1–C1 condensation in nature: acetyl-CoA synthase (ACS) and glycine synthase (GS). They are the key enzymes of the reductive acetyl-CoA pathway and the reductive glycine pathway, respectively.^15,16^ GS and ACS are structurally and mechanistically highly complex biocatalysts (the latter being limited to strictly anaerobic conditions),^17,18^ which illustrates the challenge of direct C1–C1 condensations and their implementation in microbial platform organisms. Thus, the discovery or development of simpler C1–C1 carboligases could enable new-to-nature linear carbon fixation pathways and ultimately facilitate the engineering of whole-cell catalysts for efficient C1 conversions.^7,19^

One way to generate nucleophilic (one)-carbon centers is to invert the reactivity of a carbonyl species through Umpolung, which enzymes can achieve through the cofactor thiamine diphosphate (ThDP).^20^ In recent years, several ThDP-dependent enzymes have been engineered to catalyze C1-extension reactions, which underscores the general potential of these enzymes for synthetic carbon fixation pathways. The most prominent example is the new-to-nature enzyme formolase (FLS), which was derived from benzaldehyde lyase.^21^ Similarly, glycolaldehyde synthase (GLS) was engineered from benzoylformate decarboxylase.^22^ Although in both cases a very low initial activity was improved ∼100-fold by directed evolution, the final enzyme variants still exhibited low catalytic efficiencies (*k*_cat_/*K*_M_ < 10 M^–1^ s^–1^), as well as poor affinity for highly toxic formaldehyde (*K*_M_ ≥ 170 mM). While these engineered enzymes indeed were able to support new-to-nature linear C1 assimilation pathways in vitro, the low substrate affinity and activity limited their implementation in vivo so far.^21,22^

**Figure 1:**
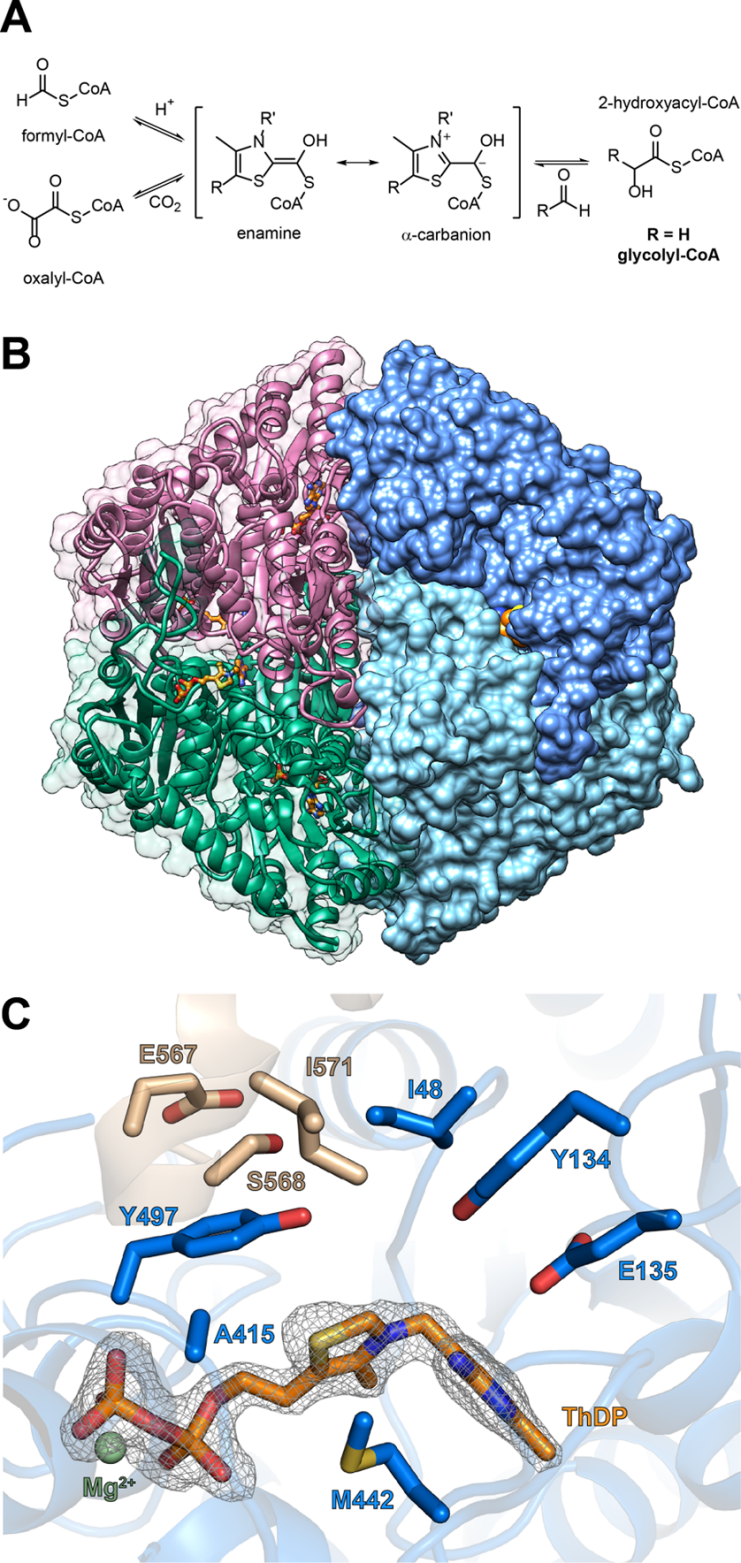
Crystal structure of MeOXC reveals amino acids surrounding the ThDP cofactor as potential targets for engineering the GCS activity. (A) Reaction scheme of OXC and HACL. The reaction proceeds via the α-hydroxyl-CoA-ThDP covalent intermediate on the ThDP cofactor, which has an enamine and an α-carbanion resonance structure. The intermediate can perform a nucleophilic attack on an aldehyde, giving rise to 2-hydroxyacyl-CoA (glycolyl-CoA in the case of the GCS reaction). (B) The overall structure of MeOXC with bound ThDP (orange). Shown is a homotetramer. (C) Amino acids of MeOXC (blue) in the environment of ThDP (orange). The C-terminal loop of the OfOXC structure is shown in light brown. The simulated annealing omit map around the refined ThDP is shown at σ = 4. The amino acids I48, E134, Y135, A415, Y497, S568, E567, and I571 were selected for saturation mutagenesis, based on their probable proximity to the α-carbanion/enamine intermediate (see A and Figure S2).

We recently identified another class of ThDP-dependent enzymes as potential formaldehyde carboligases. The members of the 2-hydroxyacyl-CoA lyase (HACL)/oxalyl-CoA decarboxylase (OXC) enzyme family catalyze the condensation of formyl-CoA with formaldehyde to produce glycolyl-CoA. This activity will be referred to as glycolyl-CoA synthase (GCS) (Figure 1A).^23,24^ Notably, HACL from *Rhodospirillales bacteriumURHD0017* (RuHACL) displayed more than 10-fold higher catalytic efficiency (*k*_cat_/*K*_M_ = 110 M^–1^ s–1) compared to the engineered FLS and GLS. However, efforts of further engineering HACL toward higher GCS activity have not been successful,^23^ mainly because of the lack of structural information. So far, no HACL structure is available and homology models fail to accurately predict the structure of the C-terminal active site loop, which exhibits low sequence homology throughout the HACL/OXC family (Figure S1). Moreover, another limitation of HACLs is their poor production in *E. coli*.^23,24^

To overcome these challenges with HACLs, we recently focused on repurposing OXCs, which naturally catalyze the decarboxylation of oxalyl-CoA, as formyl-CoA condensing enzymes.^24^ Here, we aimed at improving the GCS activity of OXC to enable the production of glycolyl-CoA at high rates under physiologically relevant formaldehyde concentrations (<0.5 mM). Using structure-guided enzyme engineering, we converted OXC into a bona fide GCS through several rounds of iterative site mutagenesis (ISM)^25^ and demonstrated its function in an *E. coli* whole-cell bioconversion system to improve current production limitations and pave the way toward synthetic C1 fixation pathways.

## Results

### MeOXC Structure Identifies Targets for Mutagenesis

Previously, we showed that besides the decarboxylation of oxalyl-CoA into formyl-CoA, OXC from *Methylorubrum extorquens* (MeOXC) is also capable of catalyzing the acyloin condensation of formyl-CoA with various aldehydes, including formaldehyde.^24^ However, the catalytic efficiency of MeOXC with formaldehyde (*k*_cat_/*K*_M_ = 2 M^–1^s^–1^) is far below that of RuHACL, which is the best performing HACL known to date (*k*_cat_/*K*_M_ = 110 M^–1^s^–1^). Additionally, the *K*_M_ for both substrates is extremely high (formaldehyde: 100 mM and formyl-CoA: 3 mM; Table 1). We therefore sought to engineer MeOXC toward a more efficient GCS.

**Table 1:** Apparent Steady-State Parameters of MeOXC Variants

	formaldehyde			formyl-CoA		
enzyme	*k*_cat,app_ (s^–1^)	*K*_M,app_ (mM)	*k*_cat_/*K*_M_ (s^–1^ M^–1^)	*k*_cat,app_ (s^–1^)	*K*_M,app_ (mM)	*k*_cat_/*K*_M_ (s^–1^ M^–1^)
RuHACL^a^	3.3 ± 0.3	29 ± 8	110	1.5 ± 0.1	0.20 ± 0.05	7200
MeOXC	0.2 ± 0.01	100 ± 20	2	0.31 ± 0.08	3 ± 2	100
MeOXC1	0.34 ± 0.01	30 ± 2	10	0.23 ± 0.01	0.16 ± 0.05	1400
MeOXC2	0.57 ± 0.05	12 ± 3	50	0.43 ± 0.02	0.09 ± 0.02	5100
MeOXC3	1.6 ± 0.1	12 ± 3	130	1.6 ± 0.1	0.17 ± 0.04	9400
MeOXC4	2.0 ± 0.2	5 ± 1	400	2.4 ± 0.2	0.22 ± 0.03	11 000

a Errors reflect the standard deviation of three independent measurements. Michaelis–Menten plots are shown in Figure S7. (a) Data are taken from Chou et al.^23^

MeOXC is closely related to OXC from *Oxalobacter formigenes* (OfOXC, 63% identity) and *E. coli* (EcOXC, 61%), which both were structurally characterized .^26−28^

To gain insights into the specific active site topology of MeOXC, we solved the crystal structure of the enzyme at a resolution of 1.9 Å (Figure 1B). Overall, the structure is very similar to that of OfOXC root-mean-square deviation (rmsd = 0.425 Å for 7230 aligned atoms). Because we could not observe the electron density after residue E567, we modeled the C-terminal part (16 residues) based on the OfOXC structure (PDB ID 2ji7). In OfOXC, this part is flexible and the electron density for the closed conformation was obtained only after soaking the enzyme with the substrate or the product.^28^

During catalysis, OXC and HACL both form the same α-carbanion/enamine intermediate (Figures 1A and S2). In HACL, this intermediate is generated through proton abstraction from formyl-CoA or by cleaving off the aldehyde moiety from a 2-hydroxyacyl-CoA thioester,^29^ whereas in OXC, this intermediate is formed by decarboxylation of oxalyl-CoA.^28^ To increase the GCS activity of MeOXC, we sought to systematically alter the active site around the ThDP cofactor with ISM.^25^ Based on the crystal structure, we identified eight residues in the proximity of the ThDP cofactor as targets: I48, Y134, E135, A415, Y497, E567, S568, and I571 (Figure 1C).

### Establishing a High-Throughput Screen for GCS Activity

**Figure 2:**
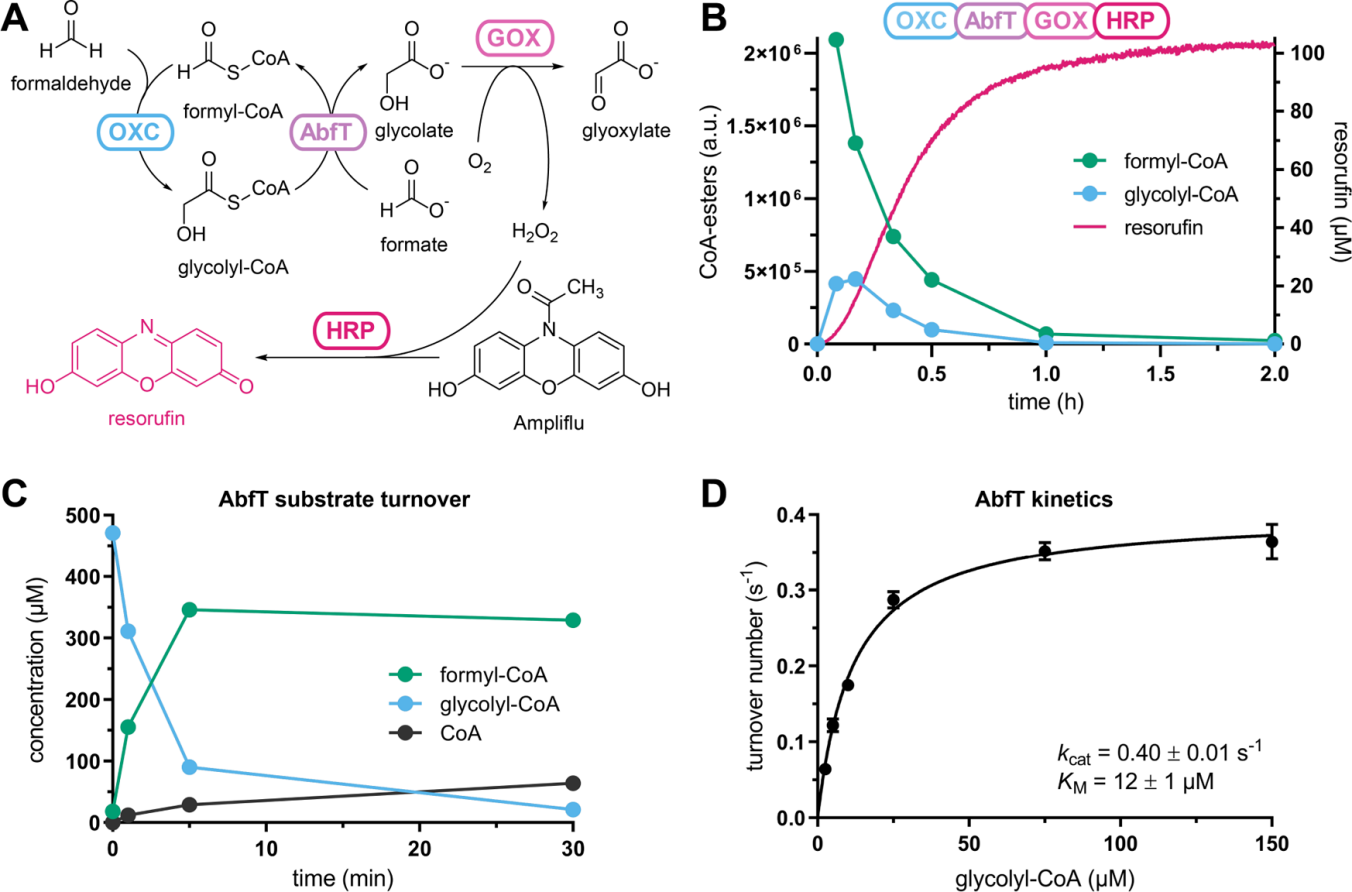
Establishing a high-throughput readout for GCS activity. (A) Reaction scheme indicating enzymes for each step. Overall, glycolyl-CoA formation is detected via the fluorophore resorufin. (B) Reaction progress of the OXC-AbfT-GOX-HRP cascade. Resorufin was detected via fluorescence in a plate reader, and CoA esters were detected by liquid chromatography-mass spectrometry (LC-MS). (C) The reaction progress of AbfT-catalyzed CoA transfer from glycolyl-CoA onto formate was monitored by LC-MS. (D) Michaelis–Menten plot of AbfT with glycolyl-CoA as a donor and formate (25 mM) as an acceptor. Error bars indicate the standard deviation of three independent replicates.

ISM requires the screening of thousands of variants.^30^ We thus conceived a high-throughput screen based on the conversion of glycolyl-CoA to glycolate, which is subsequently oxidized to glyoxylate by glycolate oxidase (GOX) under stoichiometric production of H_2_O_2_ (Figure 2A). H_2_O_2_ is quantified by horseradish peroxidase (HRP) that catalyzes the oxidation of Ampliflu Red to the fluorophore resorufin.

To convert glycolyl-CoA into glycolate, we sought to establish glycolyl-CoA:formate CoA-transferase (GFT). Such an enzyme would not only turn glycolyl-CoA into glycolate, but at the same time also regenerate formyl-CoA from formate, thereby closing a catalytic cycle for the continuous conversion of formate and formaldehyde into glycolate. We screened formyl-CoA:oxalate CoA-transferase from *Oxalobacter formigenes*(31) (FRC), propionate CoA-transferase from *Cupriavidus necactor*(32) (CnPCT), and *Clostridium propionicum*(33) (CpPCT), as well as 4-hydroxybutyrate CoA-transferase from *Clostridium aminobutyricum*(34) (AbfT). The latter showed the best GFT activity (*k*_cat,app._ = 0.40 ± 0.01 s^–1^ and *K*_M,app_(glycolyl-CoA) = 12 ± 1 μM; Figures 2C,D and S3) and was chosen for the assay.

We validated our screen with purified MeOXC, AbfT, human GOX,^35^ and HRP (Figures 2B and S4) and further demonstrated that this assay could be used to quantify the MeOXC activity from *E. coli* lysates in 384-well plates.

### Iterative Saturation Mutagenesis of MeOXC

**Figure 3:**
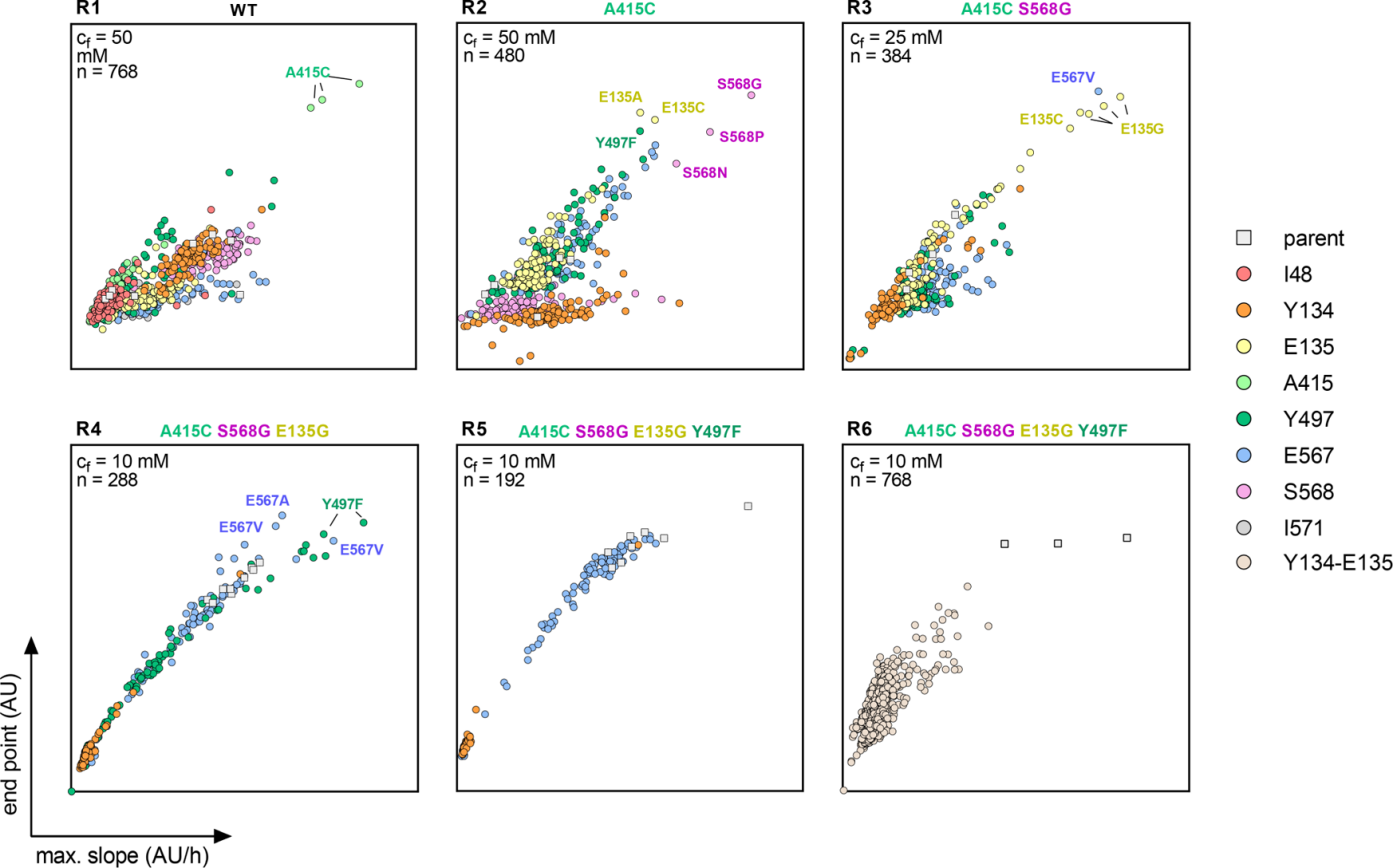
ISM of MeOXC for improved GCS activity. MeOXC saturation mutagenesis libraries were screened for GCS activity using a fluorescent-based readout in cell-free extracts. Plotted is the maximal slope (AU/h) versus the endpoint (AU) of product formation. Each panel contains all libraries of the corresponding round. The best performing variants of each round were sequenced and their substitution is shown in the graphs with colored labels. The parent variant of each round is indicated above the corresponding panel and was included in the screen as a reference. *c*_f_ = formaldehyde concentration in the screen, *n* = total number of screened clones per round, and AU = arbitrary units.

Having established a high-throughput screen, we created saturation mutagenesis libraries of the eight identified active site residues using the 22c trick (Figure S5).^30^ We employed subsaturating concentrations of formaldehyde and formyl-CoA (50 mM and 0.5 mM, respectively) to screen for MeOXC variants with improved GCS activity and higher affinity for both substrates. In the first round of ISM (R1), libraries of positions 48 and 571 contained only variants with reduced activity (Figure 3), suggesting that the isoleucines in both of these positions are critical for catalysis. Therefore, these residues were not screened in rounds R2–R6. The libraries of the remaining six residues mostly contained variants with WT-like activity but also some variants with significantly increased GCS activity, validating that these positions are potential targets to improve the enzyme activity (Figure 3). The best performing variant in R1 was an alanine to cysteine substitution in position 415. Steady-state kinetics with the purified enzyme showed that in the A415C variant, *K*_M_ for formaldehyde and formyl-CoA decreased 3- and 19-fold, respectively (Table 1), confirming that subsaturating formaldehyde concentrations could be used in our screens to identify enzyme variants with improved kinetics.

Based on the positive results of the first round, we continued with ISM using the best performing variant of each round as s template and saturating all other remaining sites stepwise. In R2, the substitution S568G conferred an ∼3-fold improvement in formaldehyde affinity, prompting us to decrease the formaldehyde concentration to 25 mM in R3, in which we identified E135G. For R4, we further lowered the concentration of formaldehyde to 10 mM to identify the substitution Y497F. In R5, we screened the remaining residues 567 and 134; however, no more positive hits were found (Figure 3). We then screened a library in which positions 134 and 135 were combinatorically saturated (R6; 400 possible variants), but this library also contained no improved variants (Figure 3). Thus, after screening ∼3600 clones in six rounds, we obtained the final variant MeOXC4 carrying four substitutions E135G, A415C, Y497F, and S568G.

### Carboligation Activity is Improved at the Expense of Decarboxylation Activity in MeOXC1–4

**Figure 4:**
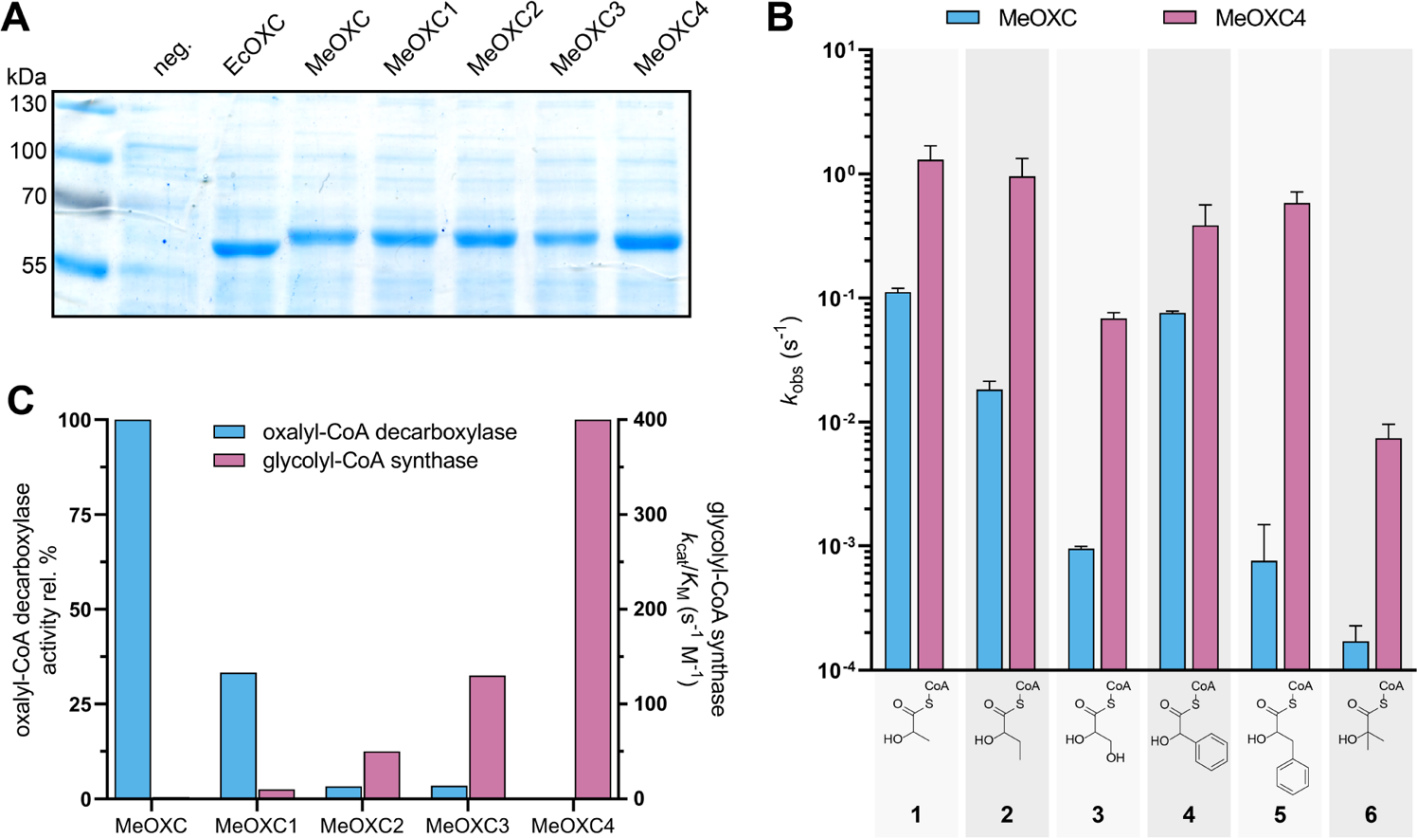
Characterization of MeOXC mutants from ISM. (A) SDS-PAGE analysis of the variants expressed in *E. coli* BL21(DE3). A total of 5 μg of the protein was loaded in each lane, and empty pET-16b was used as a negative control. EcOXC is OXC from *E. coli*. (B) Comparison of the aldehyde substrate scope of MeOXC and MeOXC4. Reactions contained 1 mM formyl-CoA and the corresponding acceptor substrate (formaldehyde, acetaldehyde, propionaldehyde, and glycolaldehyde 100 mM; benzaldehyde and phenylacetaldehyde 10 mM; and acetone 1 M). The initial velocity was determined by LC-MS. Error bars show the standard deviation of three replicates. Chemical structures of the products are shown below the graph: 1, lactyl-CoA; 2, 2-hydroxybutyryl-CoA; 3, glyceryl-CoA; 4, mandelyl-CoA; 5, 3-phenyllactyl-CoA; and 6, 2-hydroxyisobutyryl-CoA. Full data sets are shown in Figure S8. **(**C) Comparison of the OXC and GCS activities. OXC activity is the velocity of oxalyl-CoA decarboxylation at 1 mM oxalyl-CoA, relative to WT. For GCS activity, the absolute catalytic efficiency for formaldehyde is shown.

Next, we characterized MeOXC4 and the intermediate variants from R1 to R3 (MeOXC1–3) in more detail. All variants were produced in *E. coli* at levels comparable to native EcOXC (Figures 4A and S6), indicating that their improvement was based on the increased catalytic properties and not on the improved solubility and/or stability. The catalytic efficiency of the GCS activity improved over each round of ISM, ultimately resulting in 200- and 110-fold improvements in *k*_cat_/*K*_M_ for formaldehyde and formyl-CoA, respectively, due to lowered *K*_M_ for both substrates, in combination with a 10-fold increase in *k*_cat_ (Table 1).

While GCS activity strongly increased over the course of ISM, our MeOXC variants gradually lost their native OXC activity (Figure 4C).

The final variant MeOXC4 retained less than 0.2% catalytic efficiency for oxalyl-CoA decarboxylation (*k*_cat_ = 0.21 ± 0.01 s^–1^ and *K*_M_ = 122 ± 16 μM), which was exclusively due to reduced *k*_cat_, as *K*_M_ for oxalyl-CoA remained virtually identical to WT MeOXC (105 μM).^24^ This finding is in line with previous studies on OfOXC and MeOXC, where residues Y134, E135, Y497, and S568 were replaced by alanine without significant changes to *K*_M_ for oxalyl-CoA.^24,28^

Interestingly, when added to the reaction, oxalyl-CoA did not affect the GCS activity of MeOXC4 (Figure S9), even at 1 mM, indicating that the enzyme’s original substrate does not act as an inhibitor, despite still showing a very favorable apparent *K*_M_ value. This is probably due to a decreased on-rate (*k*_on_) and/or an increased off-rate (*k*_off_) for oxalyl-CoA and an opposite trend for formyl-CoA. The latter was supported by further experiments, which showed that in the presence of formaldehyde, MeOXC4 preferred carboligation and converted oxalyl-CoA after decarboxylation directly into glycolyl-CoA (*k*_obs_ = 0.15 s^–1^), releasing formyl-CoA only at a very slow rate of <0.01 s^–1^. This is in stark contrast to MeOXC WT, which shows a high formyl-CoA release rate of 98 s^–1^ after oxalyl-CoA decarboxylation, followed by slow carboligation (0.31 s^–1^) (Figure S9).

The four substitutions in MeOXC4 directly affected the catalytic activity, as well as the *k*_on_ and *k*_off_ rates of the different substrates, causing a specificity switch between native OXC (oxalyl-CoA decarboxylation) and GCS activity (formyl-CoA condensation with formaldehyde) of greater than 100 000-fold.

### Reversible (De)carboxylation Activity of MeOXC is Lost in MeOXC4

To confirm that the switch in activity was achieved by suppressing the native OXC reaction, we sought to also test the reverse reaction of OXC (i.e., the carboxylation of formyl-CoA). We envisioned an enzyme cascade in which the product of the reverse reaction, oxalyl-CoA, is constantly removed from the equilibrium by further reduction into glycolate (Figure S10A). This setup would render the overall reaction thermodynamically favorable (Δ*G* ≈ −19 kJ mol^–1^)^36^ and allow us to measure the reverse reaction, in contrast to earlier efforts, which had failed.^37^

To convert oxalyl-CoA into glyoxylate, we characterized a putative oxalyl-CoA reductase PanE2 from *M. extorquens*(38) (Figure S10B). For the reduction of glyoxylate into glycolate, we employed GhrB from *E. coli*.^39^ Combined with PanE2 and GhrB, MeOXC catalyzed the carboxylation of formyl-CoA at a rate of ∼0.5 min^–1^ (Figure S10C–E), while formyl-CoA carboxylation was not detectable in MeOXC4 (Figure S10D), confirming that this variant had indeed lost its native OXC activity.

### Structural Basis for the Improved GCS Activity in MeOXC4

To rationalize the effect of the different substitutions on catalysis, we solved the crystal structure of MeOXC4 to a resolution of 2.4 Å, modeled its flexible C-terminus beyond E567 as described before, and compared it to MeOXC WT and OfOXC.

Substitution A415C, which replaced fully conserved A415 in the HACL/OXC family by cysteine, caused a decrease in the apparent *K*_M_ value for formyl-CoA by more than an order of magnitude (Table 1). Structural analysis showed that C415 is in close proximity to S568 of the flexible C-terminal loop reaching the active site, suggesting that this substitution is important to (re-)organize the active site for the new substrate. Notably, no other amino acid substitution at this position has a beneficial effect on catalytic efficiency (Figure 3), indicating that the sulfide moiety of cysteine seems to be important for formyl-CoA accommodation. However, when testing the corresponding substitution A389C in RuHACL, GCS activity was decreased by almost 50% at subsaturating formyl-CoA concentrations (100 μM) (Figure S11), suggesting that formyl-CoA accommodation in MeOXC1–4 differs from RuHACL.

Substitution S568G lies within the flexible C-terminal loop, adjacent to A415C, and likely provides space to accommodate the bulkier side chain of C415. This is in line with the observation that S568G was only beneficial in combination with A415C and was not detected in the first ISM round (Figure 3).

**Figure 5:**
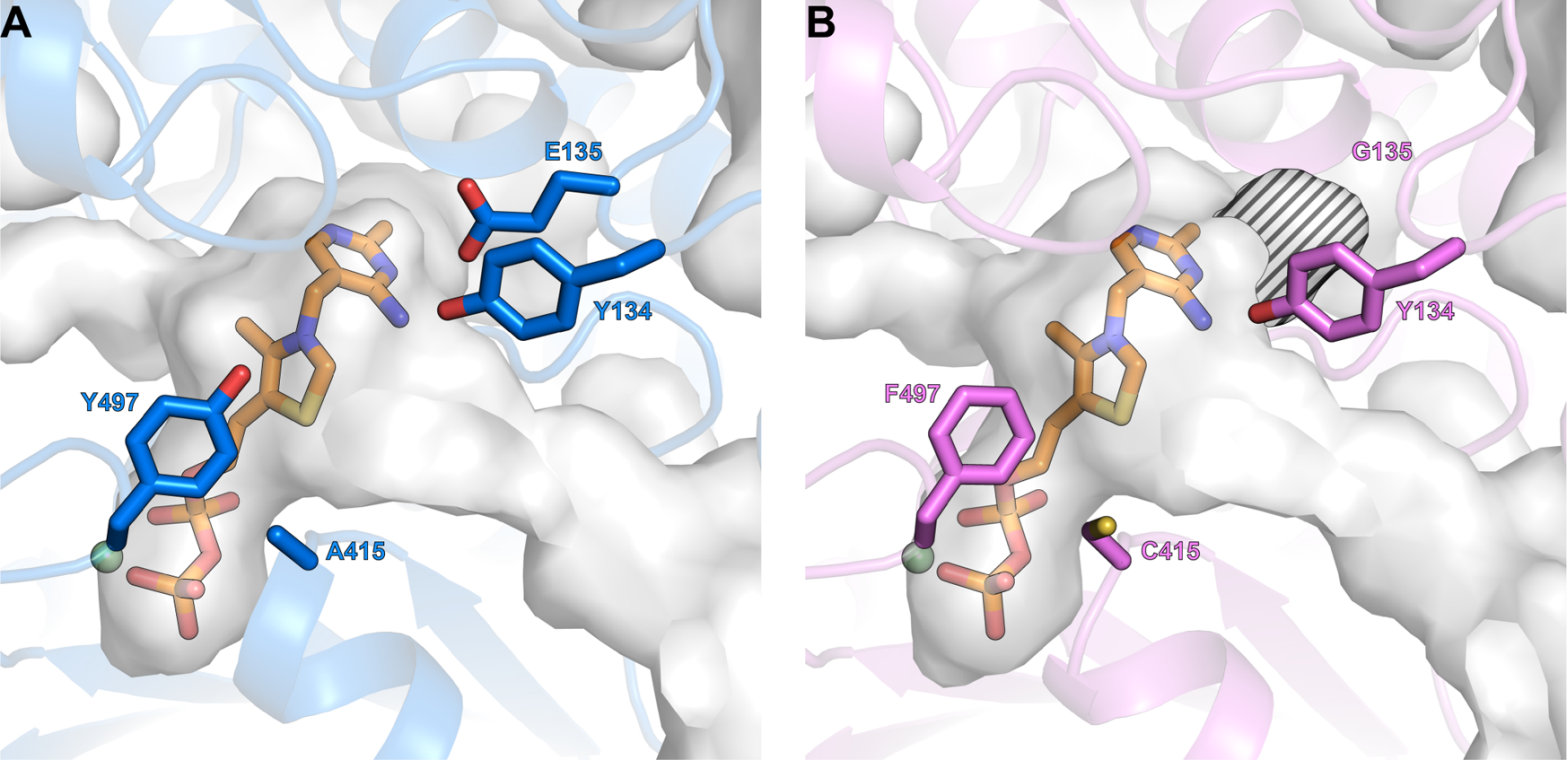
Comparison of ThDP binding sites. ThDP is shown in orange and Mg^2+^ in pale green. (A) Active site of MeOXC WT. (B) Active site of MeOXC4. Substitution E135G gave rise to a cavity in the ThDP binding pocket, highlighted with the striped pattern. Consequently, Y134 may be optimally positioned for proton transfer reactions of the glycolyl-CoA-ThDP adduct (see Figure S2).

The third substitution E135G caused the greatest structural change by creating an extra cavity at the active site (Figure 5). Notably, none of the ISM libraries at residue 134 contained a variant of improved activity (Figure 3), suggesting that Y134 is important for GCS catalysis, likely by facilitating the protonation of the glycolyl-CoA-ThDP intermediate (Figure S2). Thus, the E135G substitution does not serve in substrate accommodation but likely allows optimal positioning of Y134 during catalysis, which is supported by the fact that *k*_cat_ increased approximately fourfold by the E135G substitution, while the apparent *K*_M_ value for formyl-CoA slightly increased.

In the OfOXC active site, water molecules play a role during catalysis. Three-ordered water molecules (W1–3) are observed in a structure with a trapped α-carbanion/enamine intermediate, with W2 (in close contact to W1 and W3) proposed to protonate the Cα of the intermediate (Figure S2).^28^ W1 is hydrogen-bonded to residues corresponding to Y134 and E135, while Y497 and S568 form hydrogen bonds to W3.^28^ In MeOXC4, this hydrogen bonding network to W1 and W3 is lost due to the E135G, Y497F, and S568G substitutions. This change in the water network likely helps to promote GCS activity at the expense of the OXC reaction. In summary, our structural analysis showed that the active site of MeOXC4 is reorganized for improved substrate accommodation and increased catalytic activity.

### MeOXC4 Shows a Broad Substrate Scope for Different C1 Extensions

Having improved the catalytic efficiency of C1 extensions of formaldehyde by MeOXC4 by more than 2 orders of magnitude, we wondered whether the engineered enzyme would also promote the acyloin condensation of formyl-CoA with acceptor substrates other than formaldehyde. Indeed, MeOXC4 accepted a broad range of aldehyde substrates, including small hydrophilic and aliphatic aldehydes, bulky hydrophobic aldehydes, and acetone (Figure 4B). The activity for all tested substrates was significantly higher than that for WT MeOXC (up to a factor of 340 in the case of phenylacetaldehyde). This broad substrate scope makes MeOXC4 a versatile catalyst for the C1 extension of carbonyl acceptors that can be exploited for the biocatalytic production of valuable 2-hydroxy acids, such as lactic acid, mandelic acid, and 3-phenyllactic acid.

### Application of MeOXC4 to One-Carbon Bioconversion in *E. coli* Whole Cells

**Figure 6:**
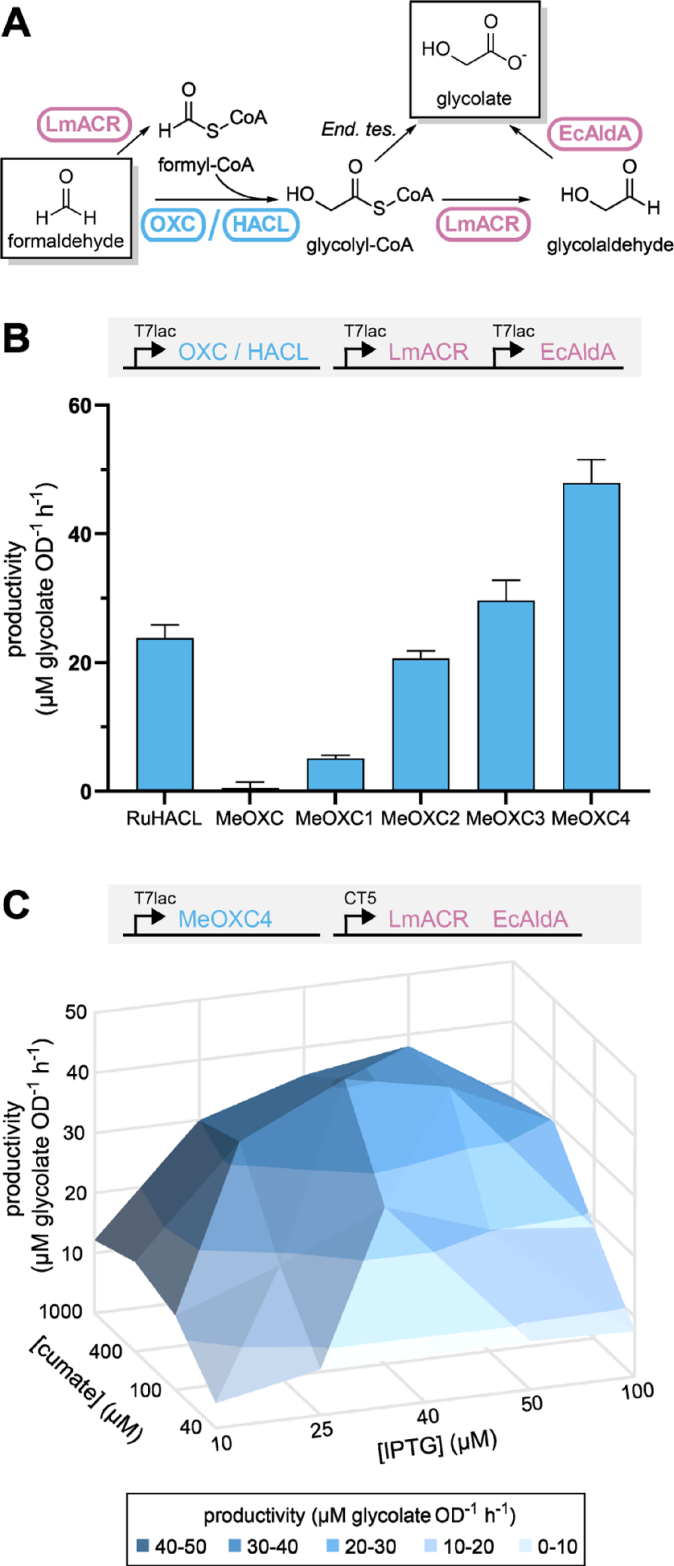
Assessing the performance of MeOXC in a synthetic one-carbon bioconversion pathway using *E. coli* whole cells. (A) The synthetic pathway converting formaldehyde to glycolate used for screening OXC/HACL variants. *End. tes*.: endogenous thioesterases. (B) The productivity of *E. coli* whole cells expressing the indicated OXC/HACL variant based on glycolate formation in 3-h bioconversions of 5 mM formaldehyde. The expression of all pathway enzymes was controlled by an IPTG-inducible T7lac promoter, with 100 μM IPTG used for induction. Bars are drawn to the mean values, and error bars represent the standard deviation of triplicate experiments. (C) Inducer concentration dependency of the productivity of *E. coli* whole cells expressing MeOXC4. Productivity is the same as defined for panel B. The expression of MeOXC4 was controlled by the IPTG-inducible T7lac promoter, while the expression of LmACR and EcAldA was controlled by a cumate-inducible T5 promoter (CT5). Mean of *n* = 4 replicates is shown as a surface plotted against IPTG and cumate concentration.

Finally, we aimed at testing the performance of our engineered enzymes also in vivo. We previously established an *Escherichia coli* whole-cell bioconversion system based on RuHACL.^23^ When combined into one pathway with an acyl-CoA reductase from *Lysteria monocytogenes* (LmACR) and *E. coli* aldehyde dehydrogenase AldA (EcAldA), whole cells converted formaldehyde into glycolate (Figure 6A). However, despite several attempts to optimize glycolate production, carbon flux was insufficient to support biotechnological applications, mainly due to the low abundance and the high apparent formaldehyde *K*_M_ value of RuHACL, which posed a major bottleneck.^23^

To test the effects of engineered MeOXC in vivo, we replaced RuHACL with variants MeOXC1–4 (Figure 6B). In line with the increasing GCS activities of the different MeOXC variants, glycolate production was successively increased. The best mutant, MeOXC4, outperformed RuHACL in glycolate productivity twofold (Figure 6B), likely because of the enzyme’s more favorable kinetics (*k*_cat_/*K*_M_ = 400 M^–1^ s^–1^ vs 110 M^–1^ s^–1^ for RuHACL) as well as improved the expression of MeOXC4 compared to RuHACL, even when the latter was codon-optimized, as confirmed for the MG1655-derived host strain (Figure S12).

These results suggested that the other enzymes of the pathway, in particular LmACR (*k*_cat_/*K*_M_ = 95 M^–1^ s^–1^),^23^ became rate limiting. To optimize the concentration of all enzymes in the pathway, we tested different expression levels, using a two-plasmid system with independent inducible promoters. MeOXC4 was expressed from an IPTG-inducible T7 promoter, while LmACR and EcAldA were coexpressed from a cumate-inducible T5 promoter (Figure 6C). Combinatorically screening of several IPTG and cumate concentrations revealed that the highest glycolate production levels were reached at low induction levels of MeOXC4 ([IPTG] = 40 μM) and high induction levels of LmACR/EcAldA ([cumate] = 400 μM) (Figure 6C). This is in contrast to our previous results, where productivity was the highest at high RuHACL and low LmACR/EcAldA induction levels.^23^ Additionally, glycolate productivity was decreased by more than 2-fold, when we replaced LmACR with a different ACR, *Rhodopseudomonas palustris* PduP,^40^ which shows a higher *k*_cat_ value but also higher *K*_M_ for formyl-CoA (Figure S13). Taken together, these results support the hypothesis that the glycolate productivity of our system is currently not limited by C1–C1 condensation but ACR activity in vivo. Thus, enhancing ACR activity will be key toward improving and further developing this C1 fixation pathway for biotechnological applications.

## Discussion

Through ISM, we successfully evolved OXC from *M. extorquens* into a bona fide GCS by switching the decarboxylation and carboligation activities of the enzyme by 100 000-fold (Figure 4C). Notably, none of the newly introduced amino acids of MeOXC4 is found in any HACL homolog (Figure S1), indicating that through directed evolution, an alternative (presumably local) maximum in the GCS activity landscape was found. This raises the question how the reactivity of OXC and HACL is determined.

It has been proposed that in OXC after decarboxylation, the α-carbanion/enamine intermediate is nonplanar, rendering the Cα more basic and facilitating the rate-limiting protonation step, yielding formyl-CoA.^28^ In contrast, an enamine-like planar structure was observed in ThDP-dependent carboligases that require a carbonyl acceptor substrate.^41−43^ It is tempting to speculate that HACLs and MeOXC4 also stabilize the enamine state of the intermediate and thereby favor nucleophilic attack on the carbonyl acceptor substrate.

Notably, engineered MeOXC4 shows kinetic parameters that are comparable with natural formaldehyde-converting biocatalysts. Only two naturally occurring enzymes are known to be involved in formaldehyde fixation, 3-hexulose-6-phosphate synthase (HPS) in the ribulose monophosphate pathway and formaldehyde transketolase (FTK) in the dihydroxyacetone pathway. The reported values of the Michaelis constant for formaldehyde range from 0.15 to 3 mM and 0.4 to 1.9 mM for HPS and FTK, respectively.^44−47^ With an apparent *K*_M_ value of 5 mM for formaldehyde, engineered MeOXC4 is fully compatible with an in vivo application, in contrast to other (engineered) C1–C1 carboligases that show apparent *K*_M_ values of 170 mM (GLS) and 29 mM (RuHACL) for formaldehyde concentrations that are toxic to *E. coli*. Compared to ACS and GS, MeOXC4 is a low-complexity C1–C1 carboligase that is homotetrameric (i.e., requires only one gene), oxygen-insensitive, and requires only ThDP and Mg^2+^ as cofactors. Additionally, MeOXC4 can be produced at high levels in *E. coli*, which makes it a versatile tool for C1 extensions. Taken together, our results highlight the potential of enzyme engineering to create new-to-nature C1 enzymes and pathways for sustainable (bio)catalysis and biotechnology.

## Supporting Information Available

Materials and Methods; Strains and plasmids used in this study (Table S1); primers used in this study. N = A, C, G, T. D = A, G, T. H = A, C, T. V = A, C, G (Table S2); data collection and refinement statistics (Table S3); concentrations of substrates and enzymes in determination of GCS steady-state parameters of MeOXC variants (Table S4); representative MSA of the HACL/OXC enzyme family (Figure S1); proposed catalytic cycle of GCS (Figure S2); screening CoA-transferases for GFT activity (Figure S3); validation of the GCS screen (Figure S4); sequencing chromatograms (Figure S5); SDS-PAGE of purified MeOXC variants (Figure S6); Michaelis–Menten graphs of MeOXC variants (Figure S7); aldehyde condensation reactions of MeOXC and MeOXC4 (Figure S8); effect of oxalyl-CoA on GCS activity of MeOXC4 (Figure S9); reverse reaction of OXC (Figure S10); comparing the GCS-activity of RuHACL G390N (WT) and RuHACL A389C G390N (Figure S11); expression analysis of formaldehyde to glycolate conversion pathway enzymes (Figure S12); effect of PduP as an ACR on the whole-cell conversion of formaldehyde to glycolate (Figure S13) (PDF)

## Abbreviations Used

CoA: coenzyme A
LC-MS: liquid chromatography-mass spectrometry
rmsd: root-mean-square deviation
