# Supplementary material for: Engineering a Highly Efficient Carboligase for Synthetic One-Carbon Metabolism
Source: ACS Catal. 2021 Apr 20;11(9):5396–404. doi: 10.1021/acscatal.1c01237 (PMC8411744; doi:10.1021/acscatal.1c01237)
Supplement: Supplementary file 1 — cs1c01237_si_001.docx [file cs1c01237_si_001.docx]

Supporting Information

Engineering a highly efficient carboligase for synthetic one-carbon metabolism

Maren Nattermann^1,‡^, Simon Burgener^1, ‡^, Pascal Pfister^1^, Alexander Chou^2^, Luca Schulz^1^, Seung Hwan Lee^2^, Nicole Paczia^1^, Jan Zarzycki^1^, Ramon Gonzalez^2^, Tobias J. Erb^1,3,*^

^1^ Department of Biochemistry & Synthetic Metabolism, Max-Planck-Institute for terrestrial Microbiology, Karl-von-Frisch-Str. 10, 35043 Marburg, Germany

^2^ Department of Chemical and Biomedical Engineering, University of South Florida, Tampa, FL, USA

^3^ LOEWE Center for Synthetic Microbiology, 35043 Marburg, Germany

* toerb@mpi-marburg.mpg.de

Contents

[Materials & Methods 4](#_Toc68168127)

[Chemicals 4](#_Toc68168128)

[Cloning and Mutagenesis 4](#_Toc68168129)

[Protein Production and Purification 4](#_Toc68168130)

[SDS-PAGE analysis of OXC expression 5](#_Toc68168131)

[Crystallization & Structure Determination 5](#_Toc68168132)

[LC-MS/MS analysis of CoA-esters 5](#_Toc68168133)

[LC-MS/MS analysis of glycolate and glyoxylate 5](#_Toc68168134)

[GFT screen 5](#_Toc68168135)

[ISM of MeOXC 5](#_Toc68168136)

[GCS kinetics 6](#_Toc68168137)

[Aldehyde scope of MeOXC vs MeOXC4 6](#_Toc68168138)

[Inhibition of MeOXC4 by oxalyl-CoA 6](#_Toc68168139)

[Spectrophotometric enzyme assays 6](#_Toc68168140)

[*E. coli* whole-cell bioconversions 7](#_Toc68168141)

[Supporting Tables 8](#_Toc68168142)

[Table S1. 8](#_Toc68168143)

[Table S2. 9](#_Toc68168144)

[Table S3.  10](#_Toc68168145)

[Table S4. 11](#_Toc68168146)

[Supporting Figures 12](#_Toc68168147)

[Figure S1. Representative MSA of the HACL/OXC enzyme family. 12](#_Toc68168148)

[Figure S2. Proposed catalytic cycle of GCS. 13](#_Toc68168149)

[Figure S3. Screening CoA-transferases for GFT activity. 14](#_Toc68168150)

[Figure S4. Validation of the GCS screen. 15](#_Toc68168151)

[Figure S5. Sequencing chromatograms of the 22c-trick*^10^* libraries. 16](#_Toc68168152)

[Figure S6. SDS-PAGE of purified MeOXC variants. 17](#_Toc68168153)

[Figure S7. Michaelis-Menten graphs of MeOXC variants. 18](#_Toc68168154)

[Figure S8. Aldehyde condensation reactions of MeOXC and MeOXC4. 19](#_Toc68168155)

[Figure S9. Effect of oxalyl-CoA on GCS activity of MeOXC4. 20](#_Toc68168156)

[Figure S10. Reverse reaction of OXC. 21](#_Toc68168157)

[Figure S11. Comparing the GCS-activity of RuHACL G390N (WT) and RuHACL A389C G390N. 22](#_Toc68168158)

[Figure S12. Expression analysis of formaldehyde to glycolate conversion pathway enzymes. 23](#_Toc68168159)

[Figure S13. Effect of PduP as an ACR on the whole-cell conversion of formaldehyde to glycolate. A) Kinetic parameters for the conversion of formaldehyde to formyl-CoA. B) 24](#_Toc68168160)

[Supporting References 25](#_Toc68168161)

# Materials & Methods

## Chemicals

Chemicals were obtained from Sigma-Aldrich (Munich, Germany) and Carl Roth GmbH (Karlsruhe, Germany). Coenzyme A was obtained from Roche Diagnostics Deutschland GmBH (Mannheim, Germany). Biochemicals, commercially available proteins and materials for cloning and protein production were obtained from Thermo Fisher Scientific (St. Leon-Rot, Germany), New England Biolabs GmbH (Frankfurt am Main, Germany) and Macherey-Nagel GmbH (Düren, Germany). Primers and synthesized genes were obtained from Eurofins MWG GmbH (Ebersberg, Germany).

Synthesis of CoA-esters

Formyl-CoA and oxalyl-CoA were synthesized and purified as described previously.*^1^* Glycolyl-CoA, lactyl-CoA, mandelyl-CoA, 3-phenyllactyl-CoA and 2-hydroxyisobutyryl-CoA were synthesized via carbonyldiimidazole coupling, as described previously*^2, 3^*. Glyceryl-CoA and 2-hydroxybutyryl-CoA were synthesized enzymatically. For glyceryl-CoA, 100 mM sodium phosphate pH 6.5, 5 mM MgCl_2_, 0.15 mM ThDP, 100 mM propionaldehyde, 10 mM formyl-CoA were mixed and the reaction was initiated by adding MeOXC Y497A*^1^* to a final concentration of 2 mg/mL. After 23 minutes at 30 °C, the reaction was quenched with formic acid (final concentration 5% (v/v)). For 2-hydroxybutyryl-CoA, 100 mM sodium phosphate pH 6.5, 5 mM MgCl_2_, 0.15 mM ThDP, 100 mM propionaldehyde, 10 mM formyl-CoA were mixed and the reaction was initiated by adding MeOXC4 to a final concentration of 2 mg/mL. After 23 minutes at 30 °C, the reaction was quenched with formic acid (final concentration 5% (v/v)).

All CoA-esters were purified by preparative HPLC-MS with a methanol gradient in 25 mM ammonium formate pH 4.2 (formyl-, glycolyl- and oxalyl-CoA) or 8.1 (glyceryl-, 2-hydroxybutyryl-, 2-hydroxyisobutyryl-, lactyl-, mandelyl-, 3-phenyllactyl-CoA). The fractions containing the product were lyophilized and stored at -20 °C.

For use in assays and standards, CoA esters were dissolved in 100 mM potassium phosphate, pH 5.5. The concentration was determined by enzymatic depletion with PduP*^4^*, following NADH consumption at 340 nm.

## Cloning and Mutagenesis

Strains and plasmids are listed in **Table S1**. Human *gox* was codon optimized and synthesized by Twist Bioscience, PCR amplified using the primers listed in **Table S2** and cloned into pET28b using NdeI and BamHI. *panE2* was PCR-amplified from *M. extorquens* chromosomal DNA using the corresponding primers (**Table S2**). The purified PCR products were digested with NdeI and BamHI and ligated into pET-16b.

Point mutations were introduced by PCR using mismatch primers (**Table S2**). A 50 µL reaction contained 60 ng of template DNA, 0.25 µM forward and reverse primer, 200 µM dNTP, 5 µL 10x Reaction Buffer, 1 µL Phusion polymerase (2 U/µL). Template plasmid was removed by DpnI digest (10 U) at 37 °C immediately after PCR amplification. Correct cloning was confirmed by sequencing (Eurofins Genomics, Ebersbach, Germany and Microsynth AG, Balgach, Switzerland)

For *E. coli* whole-cell bioconversion experiments, MeOXC and mutant variants 1-4 were PCR-amplified using primers (**Table S2**) having 15-bp homology to the destination vector, pCDFDuet-1 (Novagen). pCDFDuet-1 was linearized by restriction digest using EcoRI-HF (NEB Inc.). PCR and digestion products were gel purified and ligated using the In-Fusion HD EcoDry cloning kit (Takara Bio USA) following manufacturer directions. Resulting plasmids isolated from single colonies were verified by sequencing (Eurofins Genomics LLC). Sequence verified plasmids were further used to create bioconversion strains (**Table S1**) by electroporation using standard protocols.

## Protein Production and Purification

All proteins were heterologously produced in *E. coli* BL21 (DE3). 500 mL TB containing 100 µg/mL ampicillin (AbfT, FRC, GOX, MeOXC(1-4), PanE2, PCTs, PduP, RuHACL G390N, RuHACL A389C G390N) or 34 µg/mL chloramphenicol (GhrB) was inoculated with freshly-transformed cells and incubated at 37 °C. After reaching an OD_600_ of 0.8 expression was induced by adding IPTG to a final concentration of 0.5 mM and the incubation temperature was lowered to 25 °C. Cells were harvested after 16 h by centrifugation (4500× g, 10 min) and resuspended in buffer A (500 mM KCl, 50 mM HEPES-KOH pH 7.8). If not used immediately, cell pellets were stored at −20 °C. The cell lysate obtained by sonication was clarified by centrifugation 75,000× g at 4 °C for 45 min. The supernatant was filtered through a 0.4 µm syringe tip filter (Sarstedt, Nümbrecht, Germany). Ni-affinity purification was performed with an Äkta FPLC system from GE Healthcare (GE Healthcare, Freiburg, Germany). The filtered soluble lysate was loaded onto a 1 mL Ni-Sepharose Fast Flow column (HisTrap FF, GE Healthcare, Little Chalfont, UK) that had beenx equilibrated with 10 mL buffer A. After washing with 20 mL 85% buffer A, 15% buffer B (500 mM KCl, 50 mM HEPES-KOH pH 7.8, 500 mM imidazole), the protein was eluted with 100% buffer B. Fractions containing purified protein were pooled and the buffer was exchanged to storage buffer (150 mM KCl, 50 mM HEPES-KOH pH 7.8) with a desalting column (HiTrap, GE Healthcare). PduP was applied to a 1 ml StrepTrap column (GE Healthcare) that had been equilibrated with storage buffer. The column was washed with storage buffer and the recombinant enzyme was eluted with storage buffer containing 2.5 mM desthiobiotin. Proteins were concentrated by ultrafiltration (Amicon Ultra). Concentration was determined on a NanoDrop 2000 Spectrophotometer (Thermo Scientific, Waltham, MA, USA) using the extinction coefficient at 280 nm, as calculated by protparam (https://web.expasy.org/protparam/). Enzyme purity was confirmed by SDS-PAGE. The purified proteins were stored in 50% glycerol at −20 °C. MeOXC WT and mutants were flash-frozen in liquid nitrogen and stored at −80 °C.

## SDS-PAGE analysis of OXC expression

Protein was produced as described above. Protein concentration in the clarified cell lysate was determined with the Bradford assay, using bovine serum albumin for calibration.*^5^* 5 µg of total protein was loaded in each lane. As negative control, empty pET-16b plasmid was used and MeOXC expression was compared to EcOXC (overexpressed from plasmid pCA24N-EcOXC).

## Crystallization & Structure Determination

MeOXC and MeOXC4 were purified as described above. Immediately after affinity purification, the eluate was loaded onto a HiLoad 16/600 Superdex 200 pg column (GE Healthcare) equilibrated in SEC Buffer (75 mM KCl, 25 mM HEPES-KOH pH 7.8). Fractions corresponding to tetrameric enzyme were collected, pooled and concentrated to 10 mg/mL on 30,000 MWCO filters (Amicon Ultra). Enzyme purity was evaluated via SDS page. The enzyme was supplemented with 10 mM MgCl_2_, 2 mM TPP and 1 mM CoA and crystal plates were set up using the sitting drop vapor diffusion method, diluting equal volume of enzyme in reservoir solution. For MeOXC the reservoir solution contained 45 % w/v pentaerythritol ethoxylate (3/4 EO/OH), 100 mM sodium acetate pH 4.6, 400 mM KCl); and for MeOXC4 25 % w/v pentaerythritol propoxylate (17/8 PO/OH), 100 mM TRIS pH 8.5, 50 mM MgCl_2_). Substrate soaking with glycolyl-CoA improved the final resolution of the crystals without resulting in clear electron density for these ligands.

Data for MeOXC was collected at the Beamline ID23 -1 (European Synchrotron Radiation Facility, Grenoble, France), whereas data for MeOXC4 was collected at the Beamline P13 (Deutsches Elektronen-Synchrotron, Hamburg, Germany). All images were processed using XDS.*^6^* The dataset was scaled using the program suite ccp4.*^7^* The Phenix software package*^8^* was used to perform molecular replacement (PhaserMR) for phasing of the MeOXC dataset by using the oxalyl-CoA decarboxylase from *Oxalobacter formigenes* (PDB 2C31) as search model. The refined structure of MeOXC was then used as search model for MeOXC4. Initial models were built with Phenix.AutoBuild and refined with the phenix.refine. Manual refinement and ligand modelling was done in COOT.*^9^* Final B-facor refinement and water positioning was also performed via phenix.refine.

## LC-MS/MS analysis of CoA-esters

Samples were prepared for LC-MS/MS analysis by quenching an aliquot of a reaction with formic acid (final concentration 4%) and centrifuging for 10 min at 17,000 rcf to remove precipitated proteins. CoA-esters were separated on an Agilent Infinity II 1290 HPLC system equipped with an EVO C18 column (50 × 2.1 mm, 1.7 μm particle size, 100 Å pore size) and a suitable guard column (20 × 2.1 mm, 5 μm particle size) (Phenomoenx, Torrance, CA, USA). Flow rate was constant, at 0.25 ml/min and 25 °C. Mobile phase A consisted of 50 mM ammonium formate and phase B of 99.9% methanol. 2 µl of sample were injected per run. The following steps were employed: 0 – 4.5 min 3% B, 4.5 – 5.5 min linear gradient 3 from 80% B, 5.5 – 6.5 min 80 % B, 6.5 – 7.5 min from 80 to 3 % B, 7.5 – 8.5 min 3% B. Masses were detected as follows on an Agilent 6495 ion funnel mass spectrometer in positive mode using an electrospray ionization source: ESI spray voltage 1000 V, sheath gas 400° C at 12 l/min, nebulizer pressure 20 psig, drying gas 100° C at 11 l/min. Components were identified by retention time and mass transition. Integration of chromatograms was performed using the MassHunter software (Agilent, Santa Clara, CA, USA). From the integrals, absolute concentrations were calculated from an external calibration curve prepared in sample matrix.

## LC-MS/MS analysis of glycolate and glyoxylate

Samples were prepared for analysis as described for the CoA-esters. An Agilent Infinity II 1290 HPLC system equipped with a Kinetex Evo C18 column (150 x .12mm, 100 A, 1.7 µm, Phenomenex) and a 20 X 2.1 mm guard column was used for separation. Flow rate was constant, at 0.1 ml/min and 25 °C. Mobile phase A was 0.1% formic acid in water and mobile phase B was 0.1% formic acid in methanol. 0.5 µl were injected. The mobile phase profile was as follows: 0 –5 min at 0 % B, 5 – 7 min from 0 to 100 % B; 7 – 10 min at 100 % B; 10 – 10.1 min from 100 to 0 % B; 10.1 – 20 min at 0 % B. Detection occurred in an Agilent 6470A mass spectrometer in negative mode with an electrospray ionization source. Settings were ESI spray voltage 4500 V, Nozzle Voltage at 500 V sheath gas 400° C at 11 l/min, nebulizer pressure 45 psig and drying gas 170° C at 5 l/min. Evaluation of the LC-MS/MS data was performed as described for the CoA-esters.

## GFT screen

For the initial screen, 50 mM TES-KOH pH 6.8, 100 mM sodium formate and 1 µM transferase (FRC, AbfT, CnPCT, CpPCT) were mixed in a 1.5 mL microfuge tube at 30 °C. The reaction was started by addition of 1 mM glycolyl-CoA. Samples were taken after 5 and 60 min and analyzed with the CoA ester method described above. For the second screen, 50 mM TES-KOH pH 6.8, 50 mM sodium formate and 2.5 µM transferase (AbfT, CnPCT) were mixed in a 1.5 mL microfuge tube at 30 °C. The reaction was started by addition of 0.5 mM glycolyl-CoA. Samples were taken after 0, 1, 5, 30 and 120 min and analyzed with the CoA ester method described above.

## ISM of MeOXC

ISM was performed using the 22c trick*^10^* and the primers listed in **Table S2**. Briefly, NDT, VHG and TGG forward primers were mixed at a ratio of 12:9:1 to create a forward primer mix. 50 µl PCR reactions contained 1x Q5 Buffer (NEB), 1x High GC enhancer (NEB), 0.4 mM dNTPs (Thermo Fisher), 0.8 µM forward primer mix, 0.8 µM reverse primer, 50 ng template DNA and 0.5 µl Q5 HF polymerase (NEB). Per target site, 4 50 µl PCR reactions were run in parallel. Two-step PCR Cycles were performed as follows: 30 s 98 °C, 32x [10 s 98 °C and 72 °C 6 min], final extension 72 °C 15 min, 4 °C hold. PCR product was purified using the Machery Nagel NucleoSpin Gel and PCR Clean-up kit and digested overnight at 37 °C using DpnI FD (Thermo Fisher) and the supplied buffer. Digested DNA was purified again and transformed into chemically competent DH5α cells (Invitrogen) by standard methods. After selection on LB plates supplied with ampicillin, colonies were washed from the plates, purified using the NucleoSpin Plasmid Miniprep kit (Machery Nagel) and send for sequencing (Microsynth). Library quality was evaluated from the Sanger sequencing traces (Figure S4). For the activity screen, libraries were transformed into competent *E.coli* BL21(DE3). Transformants were picked into 600 µl LB Amp_100_ in 2.0 ml 96 Deep Well Plates with V Bottom (Plate One). As a control, each plate was also inoculated with the corresponding parent variant in the *E.coli* BL21(DE3) background. The 96-well plates were sealed with Rotilabo Cell Culture Sealing Film (Carl Roth) and grown overnight at 37 °C, 180 rpm. The next day, fresh plates were inoculated using 30 µl of starter culture and again 600 µl LB Amp_100_. The master plates were supplemented with 200 µl 80% glycerol per well and stored at -80 °C. The fresh plates were grown at 37 °C, 180 rpm to OD_600_ = 0.4-0.6 and then induced by addition of 250 µM IPTG. Overexpression occurred for 16 h at 25 °C, 180 rpm. Cells were harvested by centrifugation in a Multifuge X1R (Heraeus) at 2000 g, 4 °C for 30 min. LB was removed and cell pellets were lysed by addition of 60 µl CellLytic B Cell Lysis Reagent (Sigma Aldrich) and incubation for 10 min at 20 °C, 120 rpm. Cell debris was spun down by centrifugation at 2000 g, 4 °C for 5 min. Activity assays were performed on Nunc 384 shallow well plates (Thermo Fisher) in an Infinite M PLEX plate reader (Tecan) using 100 mM K_2_HPO_4_, pH 7.5, 5 mM MgCl_2_, 150 µM ThDP, 50 mM formate, 50 – 10 mM formaldehyde, 0.5 mM formyl-CoA, 0.5 mM Ampliflu Red (Sigma Aldrich), 1 U/mL horse-radish peroxidase (Sigma Aldrich), 2.5 µM purified GOX, 1 µM purified AbfT and 20% (v/v) OXC lysate. Product formation was followed for 2 h. The maximal slope was determined via the first derivative of the production formation. To identify positive clones, end point concentration was plotted versus maximal slope. Positive clones were sequenced using the frozen master plates for re-inoculation. Improved activity of the purified enzyme mutant was confirmed using the same reaction conditions as for the library screen.

## GCS kinetics

GCS kinetics for formaldehyde were determined in reactions containing 100 mM potassium phosphate pH 6.9, 10 mM MgCl_2_, 500 µM ADP, 150 µM TPP and 1 mM formyl-CoA. Enzyme concentration was varied for each mutant and initial velocity was determined for five formaldehyde concentrations, as shown in **Table S4**. Similarly, kinetics for formyl-CoA were determined using reactions containing 100 mM potassium phosphate pH 6.9, 10 mM MgCl_2_, 500 µM ADP and 150 µM TPP. Formaldehyde and enzyme concentration were varied for each mutant to achieve saturating conditions and initial velocity was determined for six formyl-CoA concentrations as shown in **Table S4**. Reactions were incubated at 30 °C and samples were taken at 1, 2 and 5 min. Samples analysed by LC-MS as described above. Data was analysed in GraphPad Prism using a Michalis Menten fit. A gel of the purified MeOXC variants is shown in **Figure S5**. Michealis-Menten graphs are shown in **Figure S6**.

## Aldehyde scope of MeOXC vs MeOXC4

The aldehyde scope of MeOXC and MeOXC4 was evaluated via LC-MS. Reactions contained 100 mM potassium phosphate pH 6.9, 5 mM MgCl_2_, 150 µM TPP, 1 mM formyl-CoA, 10 µM MeOXC or 5 µM MeOXC4 and either 100 mM formaldehyde, acetaldehyde, propionaldehyde or glycolaldehyde, or 10 mM benzaldehyde or phenylacetaldehyde, or 1 M acetone. Reactions were incubated at 30 °C and samples were taken after 0, 0.5, 1, 2, 3 and 5 minutes and analysed via LC-MS.

## Inhibition of MeOXC4 by oxalyl-CoA

The impact of oxalyl-CoA on the GCS reaction was determined via LC-MS. Reactions contained 100 mM potassium phosphate pH 6.9, 10 mM MgCl_2_, 150 µM TPP, 1 mM formyl-CoA and/ or 1 mM oxalyl-CoA, 50 mM formaldehyde and 1 µM MeOXC4. Reactions were incubated at 30 °C and samples were taken after 0, 0.5, 1, 2, 3 and 5 minutes and analysed via LC-MS.

## Spectrophotometric enzyme assays

Spectrophotometric assays were performed on a Cary-60 UV/Vis spectrophotometer (Agilent) at 30°C using quartz cuvettes (10 mm path length; Hellma). For the determination of steady-state kinetic parameters, each concentration was measured in triplicates and the obtained curves were fit using GraphPad Prism 7. The data was fit to the Michaelis-Menten equation to obtain *k*_cat_ and *K*_M_ values.

**AbfT.** For the glycolyl-CoA:formate transferase activity of AbfT an assay containing 50 mM MES-KOH pH 6.8, 5 mM disodium oxalate, 50 mM sodium formate, 0.3 mM NADPH, 1.5 µM FRC, 0.2 µM PanE2 and 2 µM AbfT was preincubated for 2 min and the reaction started by adding glycolyl-CoA (2.5, 5, 10, 25, 100 and 150 µM). Reaction procedure was monitored by following the oxidation of NADPH at 340 nm.

**MeOXC forward.** The oxalyl-CoA decarboxylase activity was measured as described previously.*^1^* 50 mM MES-KOH pH 6.5, 0.3 mM NADH, 10 mM MgCl_2_, 0.5 mM ADP, 0.15 mM TPP, 5 µM PduP, and OXC (concentration depending on the mutant) was preincubated for 2 min and the reaction started by adding oxalyl-CoA (concentrations depending on the mutant). Reaction procedure was monitored by following the oxidation of NADH at 340 nm.

**MeOXC reverse.** The reverse reaction of OXC was monitored by mixing 50 mM potassium phosphate pH 6.5, 100 mM NaHCO_3_, 0.3 mM NADPH, 5 mM MgCl_2_, 0.5 mM ADP, 0.15 mM TPP, 1 mM formyl-CoA, 1µg/ml carbonic anhydrase, 0.6 µM PanE2, 2 µM GhrB, 6.6 µM OXC. The reaction was started by adding OXC and formyl-CoA, respectively, and activity monitored by following the oxidation of NADPH at 340 nm. Activity only occurred after addition of the last component.

**PanE2.** For the oxalyl-CoA reductase activity of PanE2 an assay containing 100 mM potassium phosphate pH 7.5, 0.5 mM NADPH, 18 nM PanE2 was preincubated for 2 min and the reaction started by adding oxalyl‑CoA to a final concentration of 5, 12.5, 25, 100 and 250 µM, respectively. Reaction procedure was monitored by following the oxidation of NADPH at 365 nm (ε_365nm_ = 3.4 mM^-1^ cm^-1^).

**PduP.** Kinetic parameters for the activation of formaldehyde to formyl-CoA by PduP were determined. Reactions contained 50 mM MOPS-KOH pH 7.8, 2.5 mM NAD^+^, 1 mM CoA and 180 nM PduP. Reactions were pre-incubated for 2 minutes and started by addition of formaldehyde. Assays were performed at 30 °C. Reaction velocity was monitored as reduction of NAD^+^ at 340 nm.

## *E. coli* whole-cell bioconversions

*E. coli* strains were pre-grown in a media containing M9 basal salts (6.78 g/L Na_2_HPO_4_, 3 g/L KH_2_PO_4_, 1 g/L NH_4_Cl, 0.5 g/L NaCl, 2 mM MgSO_4_, and 15 µM thiamine-HCl) additionally supplemented with 20 g/L glycerol, 10 g/L tryptone, 5 g/L yeast extract, 50 µg/mL carbenicillin, 50 µg/mL spectinomycin, and the micronutrient solution of Neidhardt.*^11^* To test RuHACL and MeOXC variants, colonies of each strain were picked from LB-agar plates (with appropriate antibiotics) and used to inoculate 200 µL of the above media in a 2 mL deep, square 96-well plate (PlateOne). To test varying inducer concentrations, the strain expressing MeOXC4 was pre-cultured overnight in LB media containing appropriate antibiotics and used to inoculate an appropriate volume of the above media (1% inoculation). 200 µL of the inoculated media was then distributed into the wells of a 2 mL deep, square 96-well plate. Plates containing inoculated media were covered with a rayon microporous film (USA Scientific) and incubated in an incubating microplate shaker (VWR International) at 30°C and 1000 rpm. After 2.5 hours, chemical inducers (IPTG and cumate) were added in the indicated concentrations.24 hours after inoculation, cells were pelleted by centrifugation (3220×g, 8 min) and the pellet washed with 1 mL of the M9 basal salt media with the micronutrient solution of Neidhardt and without other additional supplements. Cells were pelleted again by centrifugation (3220×g, 8 min) and the cell pellets were resuspended in 1 mL of the M9 basal salt media supplemented with the micronutrient solution of Neidhardt and 5 mM formaldehyde. The plates were covered and returned to the incubating microplate shaker (30°C, 1000 rpm). After 3 hours, final OD600 of the cell suspension was measured using a Synergy H1 microplate reader (Biotek) by transferring 50 µL of cells to a 96 well plate with 50 µL media in each well for dilution. Wells containing 100 µL media were used as blanks. The deep-well plates were centrifuged (3220×g, 8 min) and glycolate in the supernatant was quantified by HPLC analysis using previously reported conditions.*^12^*

Cell pellets harvested after bioconversion were resuspended to 20OD in B-PER® Bacterial Protein Extraction Reagent (Thermo Fisher) supplemented with 0.1 mg/mL chicken egg white lysozyme (Fisher) and 5 U/mL Benzonase® nuclease (Sigma) for cell lysis. After incubation in room temperature for 15 minutes, 100 μL of each cell lysate was transferred to 1.5 mL microcentrifuge tubes for centrifugation at 15,000×g for 5 minutes. The soluble cell lysates obtained from the supernatant were analyzed using Protein 80 Kit (Agilent) by 2100 Bioanalyzer system (Agilent), following the manufacturer instructions.

# Supporting Tables

Table S1. Strains and plasmids used in this study.

| Strain/Plasmid | Description/Genotype | Source |
| --- | --- | --- |
| AC440 | MG1655 λ(DE3) ΔfrmA ΔfdhF ΔfdnG ΔfdoG ΔglcD::FRT | Chou et al.*^12^* |
| BL21 (DE3) | fhuA2 [lon] ompT gal (λ DE3) [dcm] ∆hsdS | Invitrogen |
| DH5α | fhuA2 Δ(argF-lacZ)U169 phoA glnV44 Φ80 Δ(lacZ)M15 gyrA96 recA1 relA1 endA1 thi-1 hsdR17 | Invitrogen |
| pCDFDuet-1-P1-ntH6-RuHACL^G390N^ | pCDFDuet-1 with codon optimized 6xHis-tagged *Rhodospirillales bacterium URHD0017 hacl* (Uniprot: A0A1H8YFL8) with a G390N mutation under control of the *T7lac* promoter and *lacI* | Chou et al.*^12^* |
| pCDFDuet-1-P1-ntH6-MeOXC(1-4) | pCDFDuet-1 with 6xHis-tagged *Methylorubrum extorquens* *oxc* (or indicated mutant) under control of the *T7lac* promoter and *lacI* | This study |
| pET-16b-s-PduP | pET-16b with strep-tagged *Rhodopseudomonas palustris* *pduP* under control of *T7* promoter and *lacI* | Zarzicky et al.*^4^* |
| pCA24N-GhrB | pCA24N with *Escherichia coli* *ghrB* under control of T5 promoter and *lacI* | ASKA collection*^13^* |
| pCA24N-EcOXC | pCA24N with *Escherichia coli* *oxc* under control of T5 promoter and *lacI* | ASKA collection*^13^* |
| pET-16b-AbfT | pET-16b with *Clostridium aminobutyricum* *abfT* under control of *T7* promoter and *lacI* | Scheffen et al. *^14^* |
| pSEVA581-CnPCT | pSEVA581 with *Cupriavidus necator* *pct* under control of *T7* promoter | Gifted by I. Bernhardsgrütter |
| pET-16b-CpPCT | pET-16b with *Clostridium pripionicum* *pct* under control of *T7* promoter and *lacI* | Gifted by I. Berg |
| pET-16b-FRC | pET-16b with *Oxalobacter formigenes* *frc* under control of *T7* promoter and *lacI* | Gifted by A. Bar-Even |
| pET-16b-MeOXC(1-4) | pET-16b with *Methylorubrum extorquens* *oxc* (or indicated mutant) under control of *T7* promoter and *lacI* | Burgener et al.*^1^*, this study |
| pET-16b-PanE2 | pET-16b with *Methylorubrum extorquens* *panE2* under control of *T7* promoter and *lacI* | This study |
| pET-16b-RuHACL G390N | pET-16b with *Rhodospirillales bacterium URHD0017 hacl* with a G390N mutation under control of *T7* promoter and *lacI* | Chou et al.*^12^* |
| pET-16b-RuHACL A389C G390N | pET*-*16b with *Rhodospirillales bacterium URHD0017 hacl* with the mutations A389C and G390N under control of *T7* promoter and *lacI* | This study |
| pET-28b-GOX | pET-28b with *Homo sapiens* *gox* under control of *T7* promoter and *lacI* | This study |
| pETDuet-1-P1-LmACR-P2-EcAldA | pETDuet-1 with codon optimized *Lysteria monocytogenes acr* in the P1 cloning site and *E. coli aldA* in the P2 cloning site both under control of the *T7lac* promoter and *lacI* | Chou et al.*^12^* |
| pETDuet-1-P^CT5^-LmACR-EcAldA | pETDuet-1 with codon optimized *Lysteria monocytogenes acr* and *Escherichia coli aldA* in a synthetic operon under control of the cumate inducible CT5 promoter and *cymR* | Chou et al.*^12^* |
| pETDuet-1-P^CT5^-PduP-EcAldA | pETDuet-1 with codon optimized *Rhodopseudomonas palustris* *pduP* and *Escherichia coli aldA* in a synthetic operon under control of the cumate inducible CT5 promoter and *cymR* | This study |
| pTWIST-GOX | pTWIST with codon optimized *Homo sapiens* *gox* | This study |

Table S2. Primers used in this study. N = A, C, G, T. D = A, G, T. H = A, C, T. V = A, C, G.

| Name | Nucleotide sequence |
| --- | --- |
| gox_fw | GATACATATGCTGCCGCGTCTGATCTG |
| gox_rv | GTATGGATCCGCCTGCTAGCTCTAGATTAG |
| oxc_I48_NDT | CGATCTACAACGTGCCCGGCNDTCCGATCACCGATCTCGG |
| oxc_I48_VHG | CGATCTACAACGTGCCCGGCVHGCCGATCACCGATCTCGG |
| oxc_I48_TGG | CGATCTACAACGTGCCCGGCTGGCCGATCACCGATCTCGG |
| oxc_I48_rv | GCCGGGCACGTTGTAGATCGTCTCGATGCCGTTGAGCTTG |
| oxc_A415_NDT | CGATCCTCGTCAACGAGGGTNDTAACACCCTCGATCTGGC |
| oxc_A415_VHG | CGATCCTCGTCAACGAGGGTVHGAACACCCTCGATCTGGC |
| oxc_A415_TGG | CGATCCTCGTCAACGAGGGTTGGAACACCCTCGATCTGGC |
| oxc_A415_rv | CCCTCGTTGACGAGGATCGCGTCGGGCCGCTCCTTGATG |
| oxc_Y497_NDT | CTTCAACAACAACGGCATCNDTCGCGGCACCGACACCGATC |
| oxc_Y497_VHG | CTTCAACAACAACGGCATCVHGCGCGGCACCGACACCGATC |
| oxc_Y497_TGG | CTTCAACAACAACGGCATCTGGCGCGGCACCGACACCGATC |
| oxc_Y497_rv | GATGCCGTTGTTGTTGAAGATGACGATGCAGACCGGCAGC |
| oxc_I571_NDT | CCGGCAGCGAGAGCGGCAATNDTGGCAGCCTCAACCCGCAG |
| oxc_I571_VHG | CCGGCAGCGAGAGCGGCAATVHGGGCAGCCTCAACCCGCAG |
| oxc_I571_TGG | CCGGCAGCGAGAGCGGCAATTGGGGCAGCCTCAACCCGCAG |
| oxc_I571_rv | GCCGCTCTCGCTGCCGGCGGCCGGGTCGATCTCCGCATTG |
| oxc_Y134_NDT | CGATCTGCAGCAGGGCGACNDTGAGGAGATGGACCAGC |
| oxc_Y134_VHG | CGATCTGCAGCAGGGCGACVHGGAGGAGATGGACCAGC |
| oxc_Y134_TGG | CGATCTGCAGCAGGGCGACTGGGAGGAGATGGACCAGC |
| oxc_Y134_rv | GTCGCCCTGCTGCAGATCGACGATCTCGCGCTCGGAGGAG |
| oxc_E135_NDT | GATCTGCAGCAGGGCGACTACNDTGAGATGGACCAGCTCGC |
| oxc_E135_VHG | GATCTGCAGCAGGGCGACTACVHGGAGATGGACCAGCTCGC |
| oxc_E135_TGG | GATCTGCAGCAGGGCGACTACTGGGAGATGGACCAGCTCGC |
| oxc_E135_rv | GTAGTCGCCCTGCTGCAGATCGACGATCTCGCGCTCGGAG |
| oxc_E567_NDT | CGACCCGGCCGCCGGCAGCNDTAGCGGCAATATCGGCAGC |
| oxc_E567_VHG | CGACCCGGCCGCCGGCAGCVHGAGCGGCAATATCGGCAGC |
| oxc_E567_TGG | CGACCCGGCCGCCGGCAGCTGGAGCGGCAATATCGGCAGC |
| oxc_E567_rv | GCTGCCGGCGGCCGGGTCGATCTCCGCATTGATCAGAGTC |
| oxc_S568_NDT | CCCGGCCGCCGGCAGCGAGNDTGGCAATATCGGCAGCCTC |
| oxc_S568_VHG | CCCGGCCGCCGGCAGCGAGVHGGGCAATATCGGCAGCCTC |
| oxc_S568_TGG | CCCGGCCGCCGGCAGCGAGTGGGGCAATATCGGCAGCCTC |
| oxc_S568_rv | CTCGCTGCCGGCGGCCGGGTCGATCTCCGCATTGATCAG |
| oxc_S568G_E567_NDT | CGACCCGGCCGCCGGCAGCNDTGGTGGCAATATCGGCAGC |
| oxc_S568G_E567_VHG | CGACCCGGCCGCCGGCAGCVHGGGTGGCAATATCGGCAGC |
| oxc_S568G_E567_TGG | CGACCCGGCCGCCGGCAGCTGGGGTGGCAATATCGGCAGC |
| oxc_E135G_Y134_NDT | CGATCTGCAGCAGGGCGACNDTGGTGAGATGGACCAGC |
| oxc_E135G_Y134_VHG | CGATCTGCAGCAGGGCGACVHGGGTGAGATGGACCAGC |
| oxc_E135G_Y134_TGG | CGATCTGCAGCAGGGCGACTGGGGTGAGATGGACCAGC |
| oxc_Y134E135_NDTNDT | GATCTGCAGCAGGGCGACNDTNDTGAGATGGACCAGCTCGC |
| oxc_Y134E135_NDTVHG | GATCTGCAGCAGGGCGACNDTVHGGAGATGGACCAGCTCGC |
| oxc_Y134E135_NDTTGG | GATCTGCAGCAGGGCGACNDTTGGGAGATGGACCAGCTCGC |
| oxc_Y134E135_VHGNDT | GATCTGCAGCAGGGCGACVHGNDTGAGATGGACCAGCTCGC |
| oxc_Y134E135_VHGVHG | GATCTGCAGCAGGGCGACVHGVHGGAGATGGACCAGCTCGC |
| oxc_Y134E135_VHGTGG | GATCTGCAGCAGGGCGACVHGTGGGAGATGGACCAGCTCGC |
| oxc_Y134E135_TGGNDT | GATCTGCAGCAGGGCGACTGGNDTGAGATGGACCAGCTCGC |
| oxc_Y134E135_TGGVHG | GATCTGCAGCAGGGCGACTGGVHGGAGATGGACCAGCTCGC |
| oxc_E70A_fw | CATCTCCTTCCGCCACGCGCAGAATGCGGGCAAC |
| oxc_E70A_rv | GTTGCCCGCATTCTGCGCGTGGCGGAAGGAGATG |
| panE2_fw | GCGCACATATGAGCATCGCGATCGTCG |
| panE2_rv | CAGAGGATCCTCATGCTCCCTGGATCGC |
| pNov-P1-ntH6-MeOxc-L | GCCAGGATCCGAATTCTATGACCGTCCAGGCCCA |
| pNov-P1-ntH6-MeOxc-R | CGCCGAGCTCGAATTTCACTTCTTCTTCAAGGTGCTC |
| ruHACL_A389C_fw | CCTGTCTGCTGAAGGTTGCAACACCATGGACATCG |
| ruHACL_A389C_rv | CGATGTCCATGGTGTTGCAACCTTCAGCAGACAGG |
|  |  |

Table S3.  Data collection and refinement statistics. Statistics for the highest-resolution shell are shown in parentheses.

|  | MeOXC | MeOXC4 |
| --- | --- | --- |
| Beam line | ESRF_ID23-1, Grenoble, France | DESY P13, Hamburg, Germany |
| PDB ID | 7AYG | 7B2E |
| Ligands | TPP, ADP, Mg^2+^ | TPP, ADP, Mg^2+^ |
| Wavelength (Å) | 0.972 | 0.976 |
| Resolution range (Å) | 39.11 - 1.9 (1.968 - 1.9) | 29.97 – 2.8 (2.9 - 2.2.8) |
| Space group | P 2_1_ 2_1_ 2_1_ | P 2_1_ 2_1_ 2_1_ |
| Unit cell dimensions a, b, c (Å) | 160.176, 181.798, 202.366 | 161.094, 180.337, 202.008 |
| α, β, γ (°) | 90, 90, 90 | 90, 90, 90 |
| Total reflections | 2591098 (355003) | 3098058 (454913) |
| Unique reflections | 455609 (65577) | 228390 (32969) |
| Multiplicity | 5.7 (5.4) | 13.6 (13.8) |
| Completeness (%) | 98.64 (97.25) | 99.37 (99.97) |
| Mean (I)/σ (I) | 8.82 (1.99) | 10.11 (3.85) |
| Wilson B-factor (Å^2^) | 23.99 | 28.91 |
| R_merge_ | 0.04624 (0.367) | 0.04856 (0.1611) |
| R_meas_ | 0.06539 (0.519) | 0.06867 (0.2279) |
| CC1/2 | 0.997 (0.883) | 0.998 (0.978) |
| Reflections used in refinement | 454225 (44467) | 142866 (14161) |
| Reflections used for R_free_ | 1987 (195) | 1996 (197) |
| R_work_ | 0.1765 (0.3061) | 0.2012 (0.2597) |
| R_free_ | 0.2078 (0.3227) | 0.2302 (0.3175) |
| Number of non-hydrogen atoms | 36890 | 34264 |
| macromolecules | 32548 | 32560 |
| ligands | 432 | 432 |
| solvent | 3910 | 1272 |
| Protein residues | 4392 | 4405 |
| RMS(bonds) (Å) | 0.008 | 0.006 |
| RMS(angles) (°) | 1.04 | 0.86 |
| Ramachandran favored (%) | 97.54 | 97.30 |
| Ramachandran allowed (%) | 2.27 | 2.52 |
| Ramachandran outliers (%) | 0.18 | 0.18 |
| Rotamer outliers (%) | 0.94 | 0.97 |
| Average B-factor | 27.86 | 29.30 |
| macromolecules | 27.02 | 29.39 |
| ligands | 22.52 | 26.73 |
| solvent | 35.41 | 28.00 |

Table S4. Concentrations of substrates and enzymes in determination of GCS steady-state parameters of MeOXC variants. Enz, enzyme; FALD, formaldehyde; F-CoA, formyl-CoA

|  | Formaldehyde kinetics | | | Formyl-CoA kinetics | | |
| --- | --- | --- | --- | --- | --- | --- |
|  | Enz (µM) | FALD (mM) | F-CoA (mM) | Enz. (µM) | FALD (mM) | F-CoA (mM) |
| MeOXC | 5 | 500, 200, 100, 50, 20 | 1 | 5 | 500 | 12.5, 7.5, 3.75, 2.5, 1.25, 0.5 |
| MeOXC1 | 5 | 100, 50, 20, 10, 4 | 1 | 2 | 150 | 7.5, 4.5, 2.25, 1.5, 0.75, 0.3 |
| MeOXC2 | 2.5 | 50, 20, 10, 5, 2 | 1 | 1 | 80 | 1, 0.6, 0.3, 0.2, 0.1, 0.05 |
| MeOXC3 | 1 | 50, 20, 10, 5, 2 | 1 | 1 | 35 | 1, 0.6, 0.3, 0.2, 0.1, 0.05 |
| MeOXC4 | 1 | 25, 10, 5, 2, 1 | 1 | 1 | 30 | 1, 0.6, 0.3, 0.2, 0.1, 0.05 |

# Supporting Figures

Figure S1. Representative MSA of the HACL/OXC enzyme family. Shown are the sequence regions around the amino acids that are in proximity of the ThDP in MeOXC (see **Figure 1C**). MeOXC, OfOXC and RuHACL (first, third and last from the top) are in bold. The C-terminal loop (marked in blue) is highly flexible in OXC and forms part of the active site upon substrate binding. Note that this region shows low sequence conservation.

Figure S2. Proposed catalytic cycle of GCS. Catalysis is initiated by proton abstraction from the ThDP cofactor, mediated by E70A.*^15^* This generates the carbene/ylid state of ThDP, which covalently binds formyl-CoA. Deprotonation of the α-carbon by hydroxide (forming water molecule W2) then gives rise to the α-carbanion/enamine intermediate*^15^* that subsequently performs a nucleophilic attack onto formaldehyde. The resulting intermediate (glycolyl-CoA-ThDP covalent adduct) undergoes proton transfer and is then released as glycolyl-CoA, regenerating the ThDP-carbene. The proton transfer can either proceed directly between the hydroxyl groups (green arrows) or via Y134 (pink arrows).


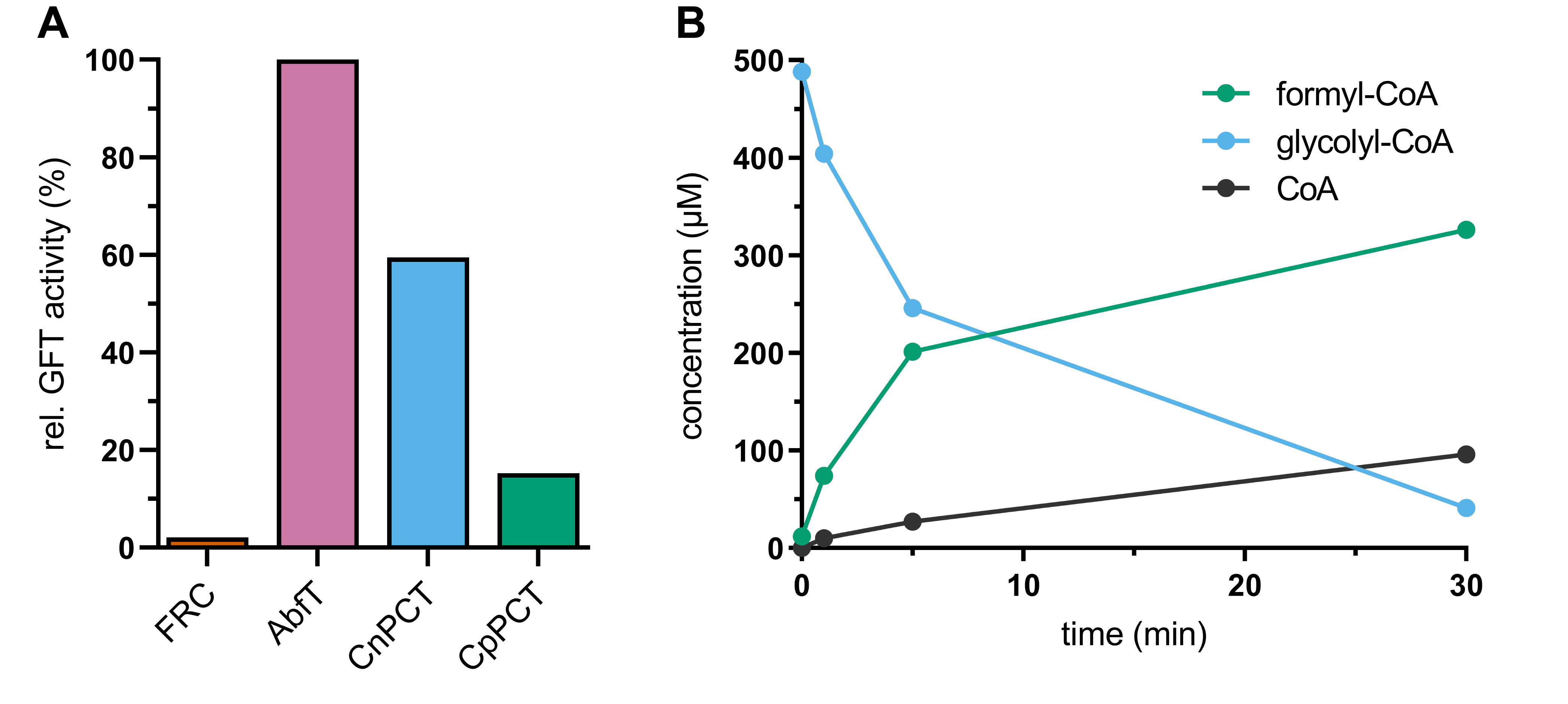


Figure S3. Screening CoA-transferases for GFT activity. **A)** GFT activity was screened by detecting the product formyl-CoA via LC-MS. Activity was normalized to the highest activity (AbfT). FRC is formyl-CoA:oxalate CoA-transferase from *Oxalobacter formigenes*, AbfT is 4-hydroxybutyrate CoA-transferase from *Clostridium aminobutyricum*, CnPCT is propionyl-CoA transferase from *Cupriavidus necator*, CpPCT is propionyl-CoA transferase from *Clostridium propionicum*. **B)** The reaction progress of CnPCT-catalyzed CoA transfer from glycolyl-CoA onto formate was monitored by LC-MS. The build-up of CoA is due to hydrolysis of formyl-CoA.


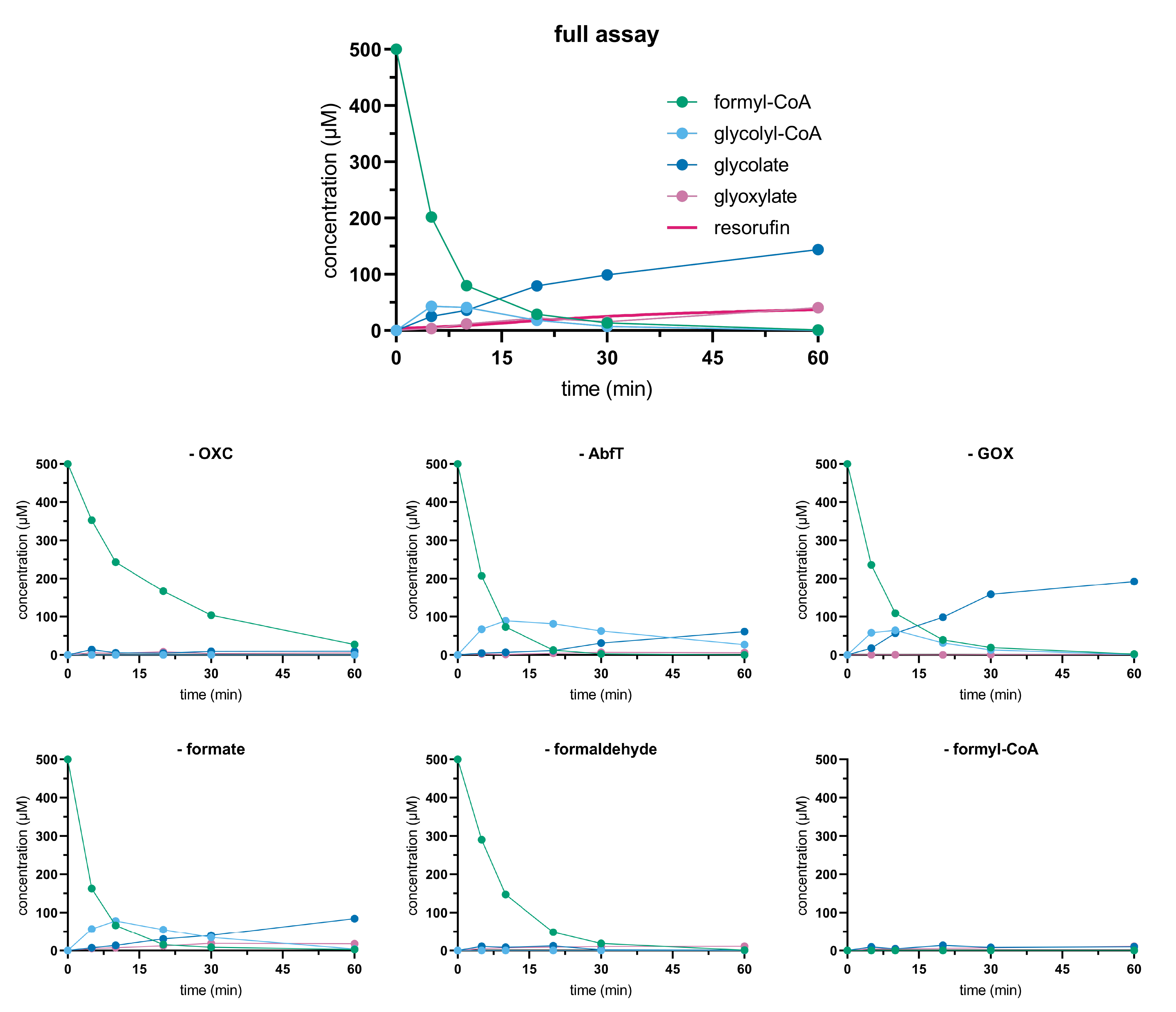


Figure S4. Validation of the GCS screen. The production of key intermediates was assessed by LC-MS and appropriate control reactions were performed to confirm the proper function of the screen. For the full assay, the complementary resorufin production was assessed by fluorescence readout in a plate reader. It was confirmed that resorufin is produced in equimolar amounts to glyoxylate.


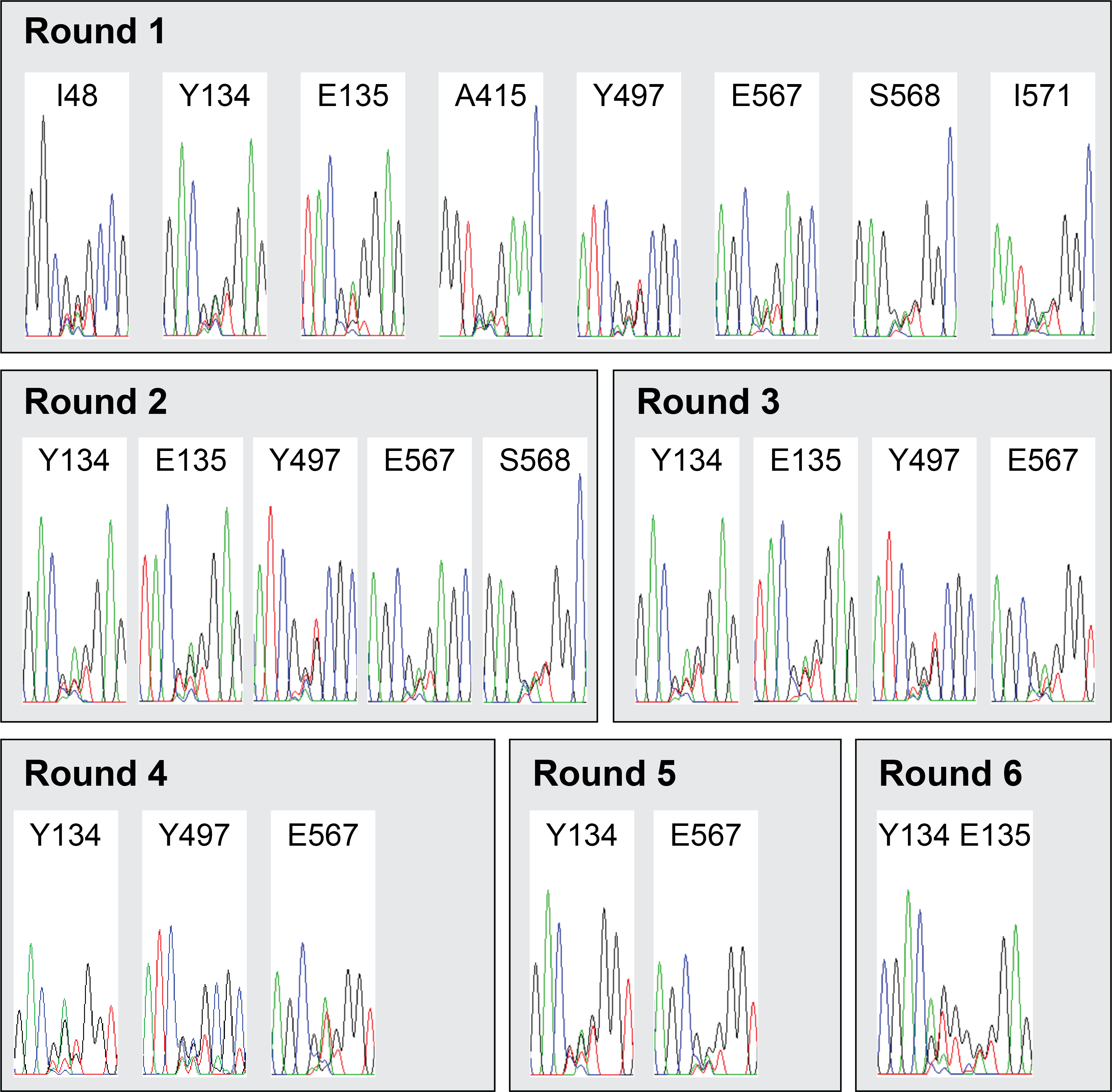


Figure S5. Sequencing chromatograms of the 22c-trick*^10^* libraries. Green, A. Blue, C. Black, G. Red, T.


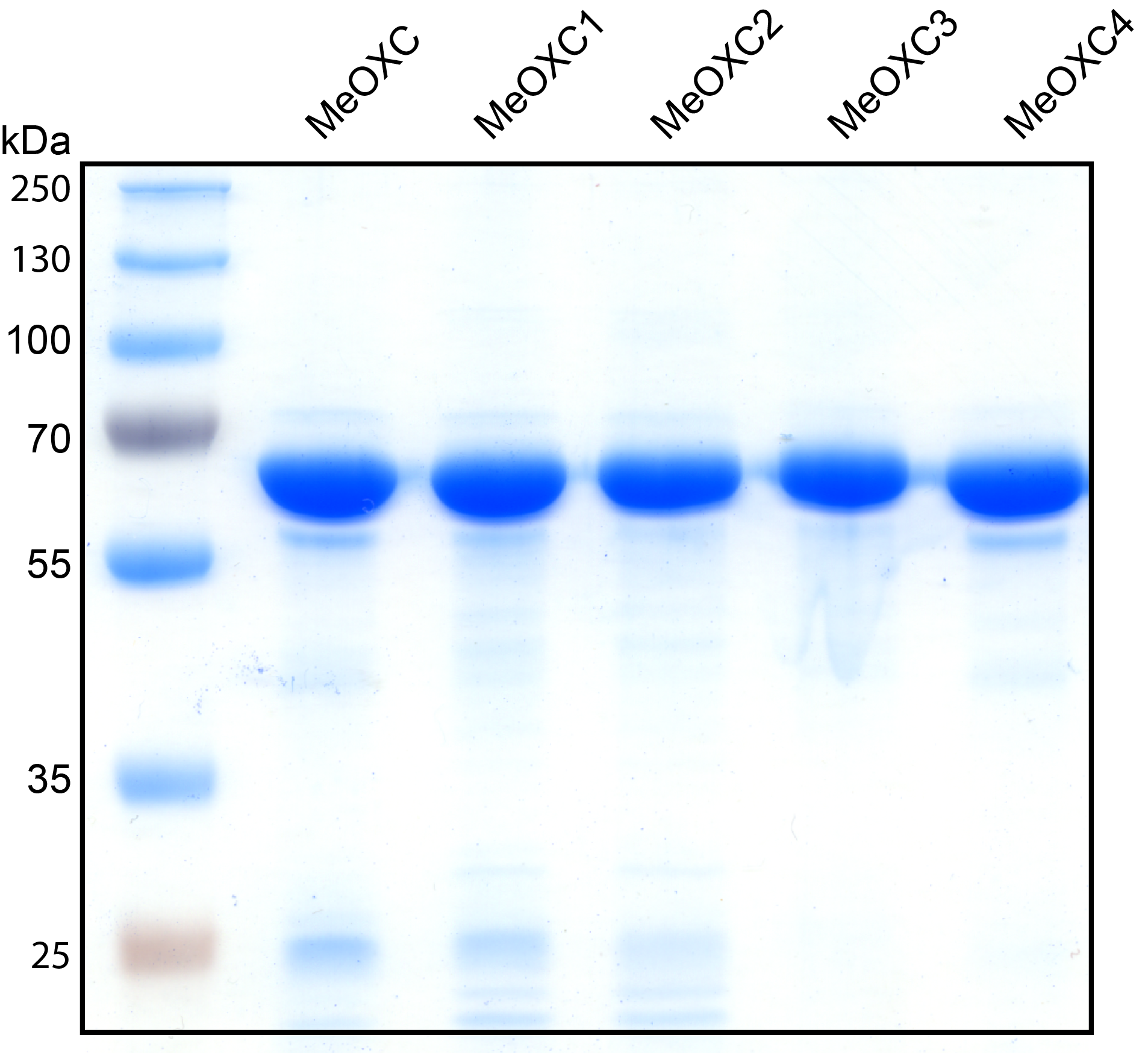


Figure S6. SDS-PAGE of purified MeOXC variants. 5 µg protein was loaded in each lane.


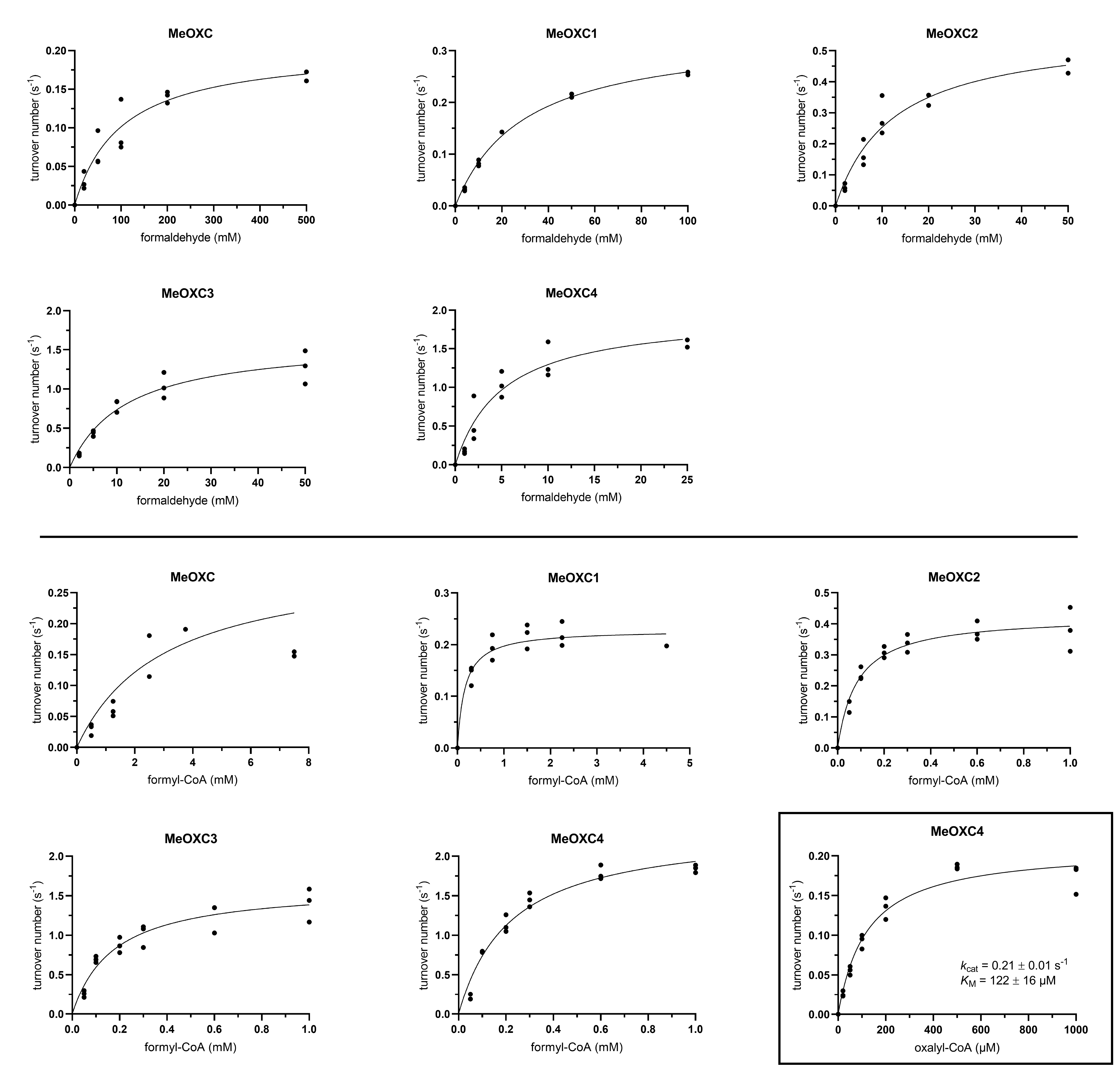


Figure S7. Michaelis-Menten graphs of MeOXC variants. Substrates are formaldehyde (top) and formyl-CoA (bottom). In the box on the bottom left is MeOXC4 with oxalyl-CoA as substrate.


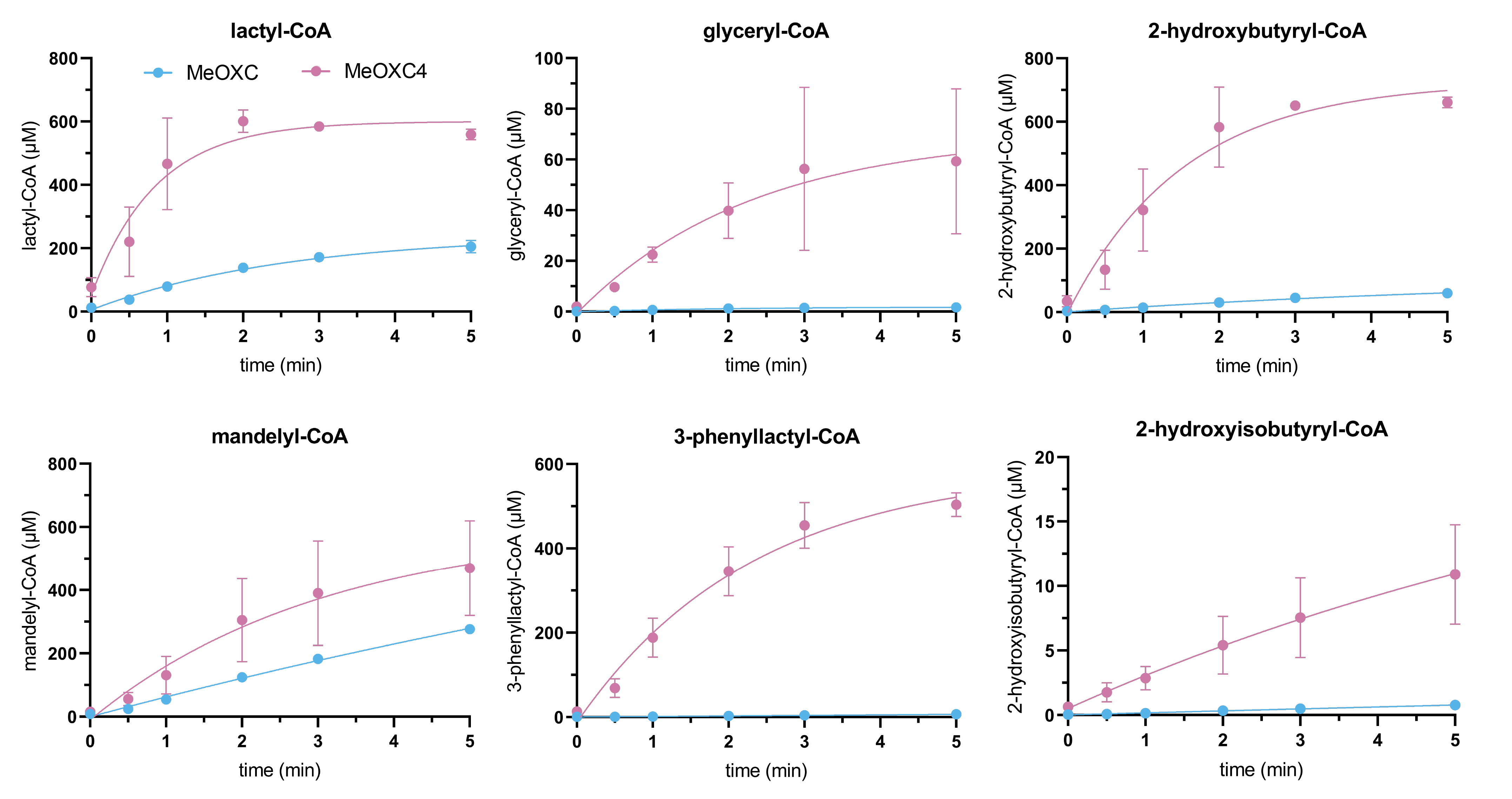


Figure S8. Aldehyde condensation reactions of MeOXC and MeOXC4. From top left to bottom right: condensation of 1 mM formyl-CoA with 100 mM acetaldehyde, 100 mM glycolaldehyde, 100 mM proprionaldehyde, 10 mM benzaldehyde, 10 mM phenylacetaldehyde and 1 M acetone. 10 µM MeOXC and 5 µM MeOXC4 were used.


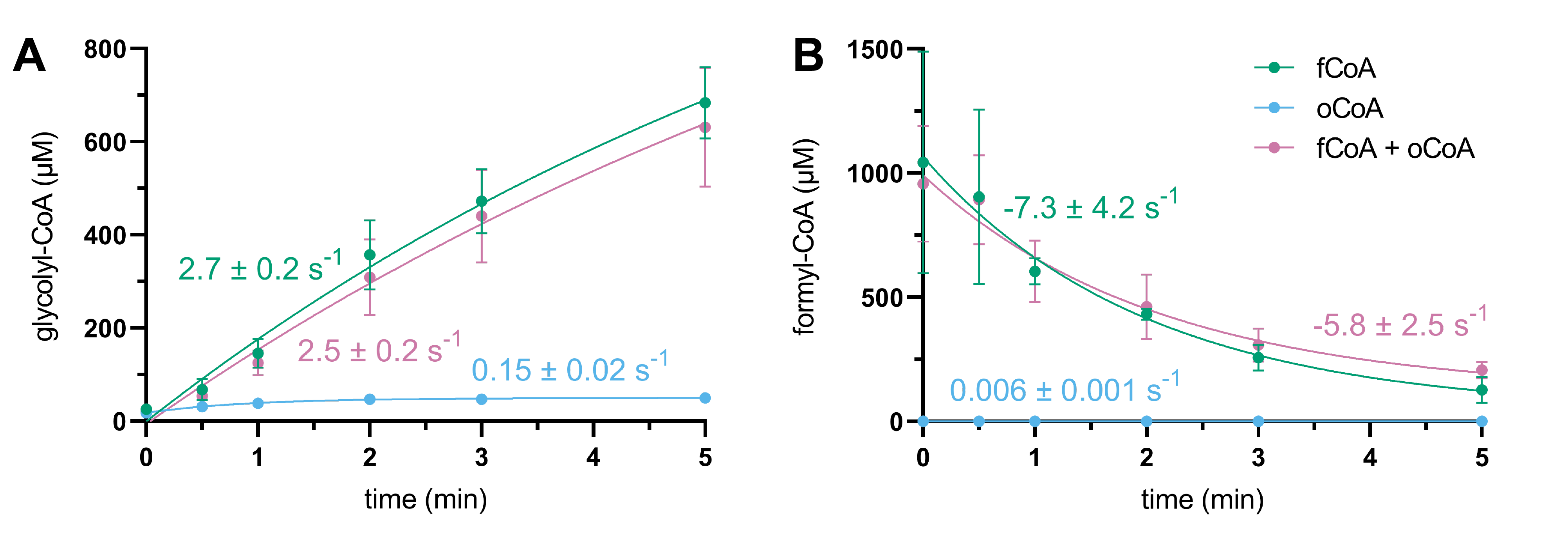


Figure S9. Effect of oxalyl-CoA on GCS activity of MeOXC4. Reactions contained 50 mM formaldehyde and 1 mM of formyl-CoA and/ or oxalyl-CoA. Error bars reflect standard deviation of three replicates. Initial velocity of each reaction is indicated (mean ± s.d.). Data is fit using a one-phase decay. fCoA, formyl-CoA; oCoA, oxalyl-CoA. **A)** glycolyl-CoA production as calculated using a standard curve. **B)** formyl-CoA consumption as estimated using the initial concentration as a calibration point.


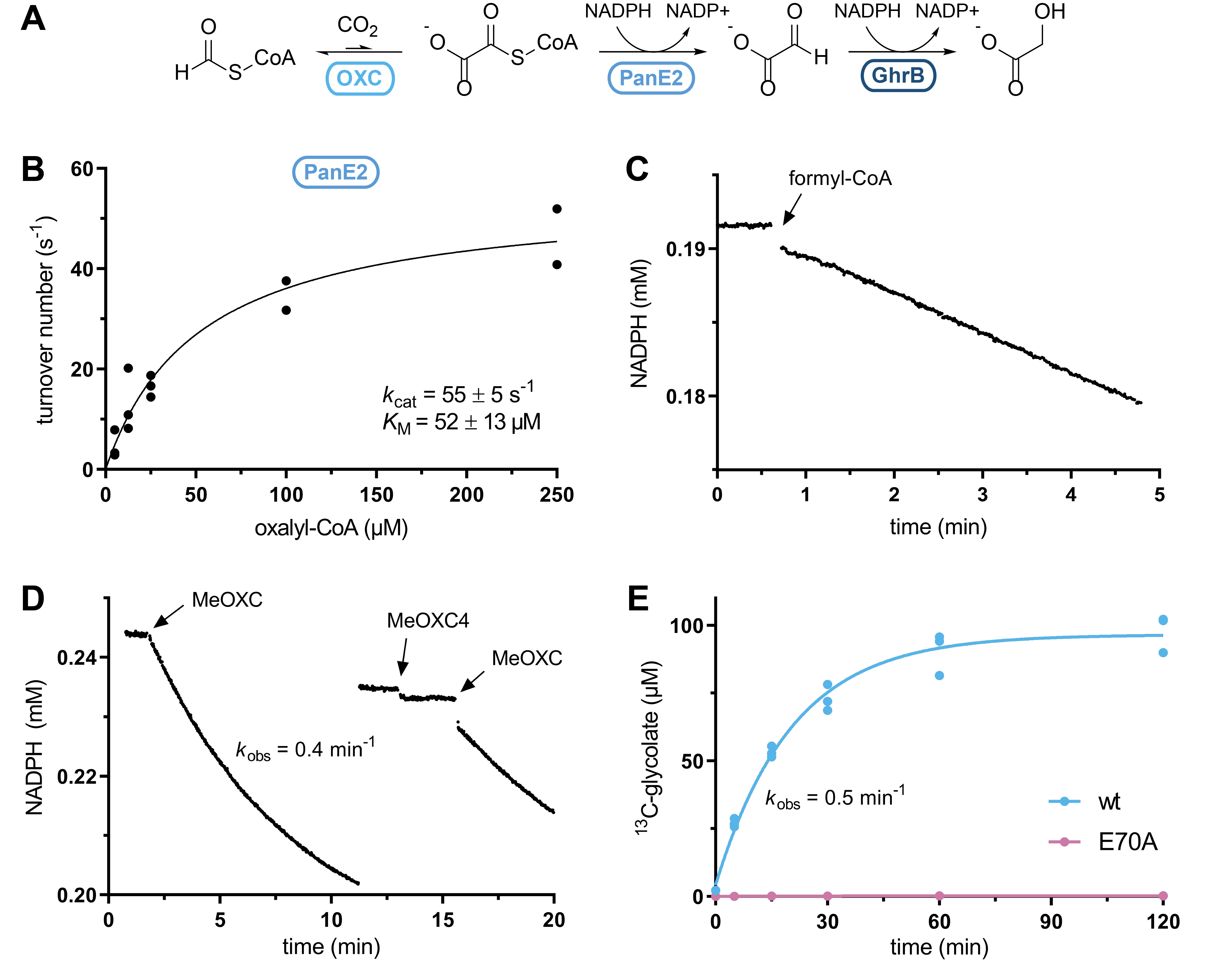


Figure S10. Reverse reaction of OXC. **A)** Reaction scheme of the OXC-PanE2-GhrB cascade, converting formyl-CoA and CO_2_ into glycolate under consumption of 2 NADPH. **B)** Michaelis-Menten plot of PanE2 catalyzing the NADPH-dependent reduction of oxalyl-CoA to glyoxylate. The NADPH concentration was 0.25 mM. Error bars indicate the standard deviation of three replicates. **C** & **D)** Operation of the full cascade was monitored by NADPH oxidation (340 nm). The arrows indicate the addition of formyl-CoA (**C**), or MeOXC (**D**). **E)** LC-MS detection of ^13^C-glycolate produced in the cascade reaction with WT OXC or the mutant E70A, which is catalytically inactive. That E70A does not show any product formation confirms that the reaction is not catalyzed by free or unspecifically bound ThDP. The *k*_obs_ values in **D** & **E** were estimated based on the initial slope of product formation.


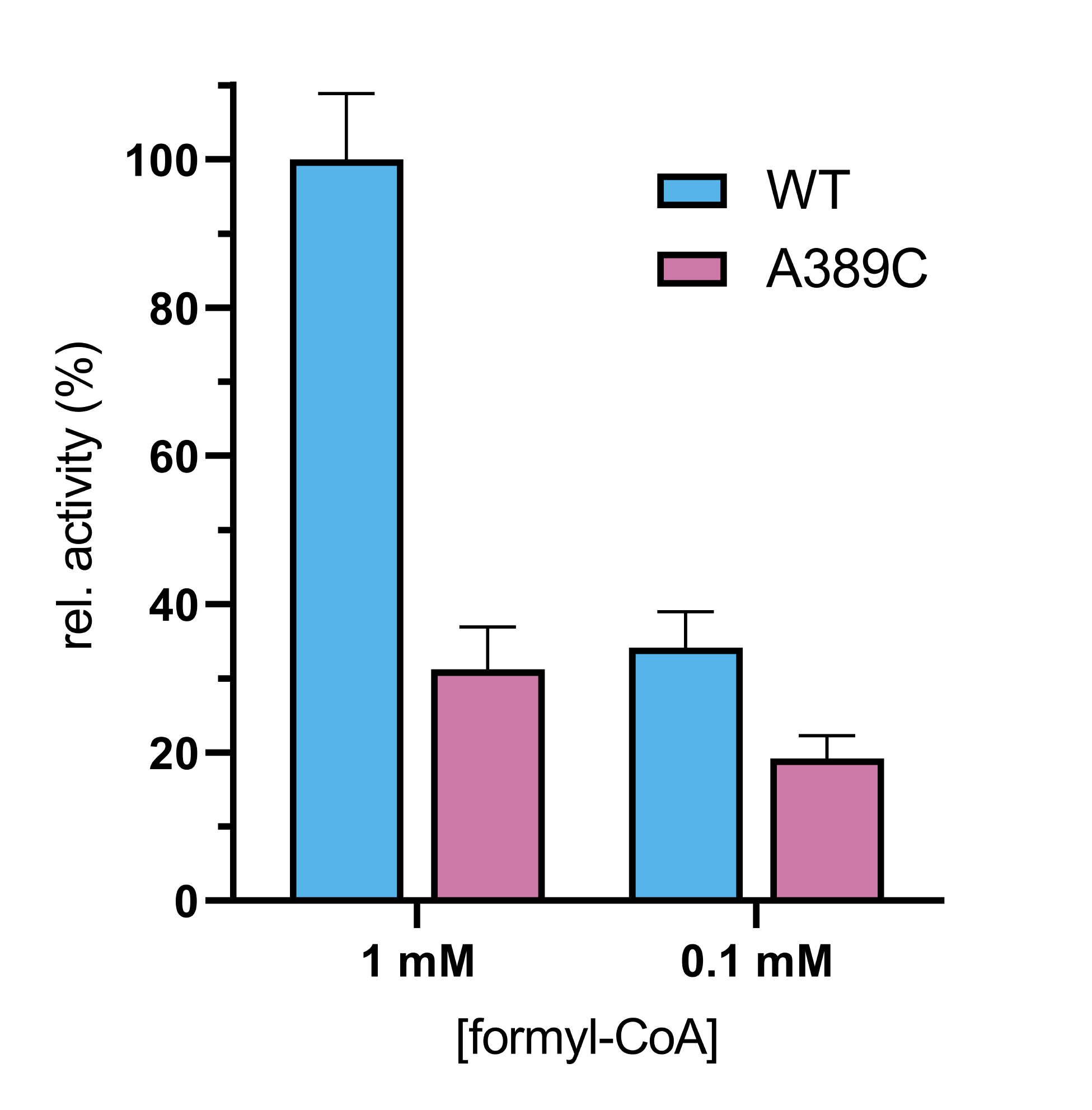


Figure S11. Comparing the GCS-activity of RuHACL G390N (WT) and RuHACL A389C G390N. The mutation A389C corresponds to MeOXC A415C. Formaldehyde concentration was 100 mM and the activity was determined by measuring glycolyl-CoA formation via LC-MS. Activity is shown relative to WT at 1 mM formyl-CoA. Error bars reflect standard deviation of three replicates.


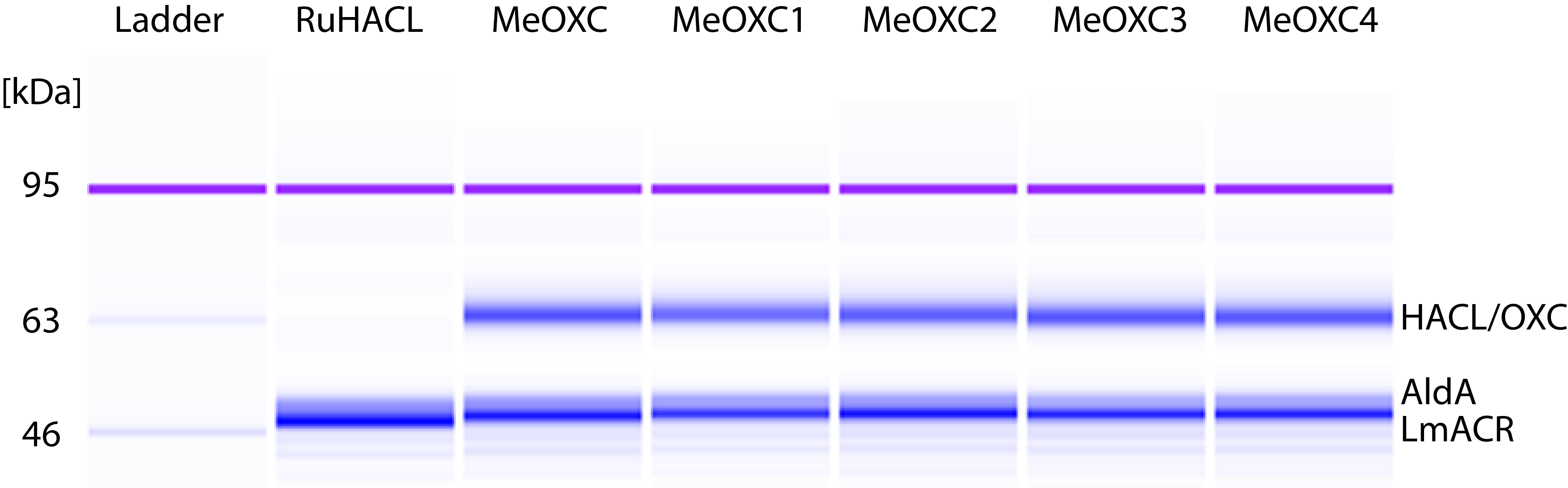


Figure S12. Expression analysis of formaldehyde to glycolate conversion pathway enzymes. RuHACL (~61 kDa) expresses at a substantially lower level than MeOXC variants (~63.5 kDa) in strain AC440, derived from *E. coli* MG1655. The gel representation was generated by 2100 Bioanalyzer Expert software (Agilent) with intensity normalized on a per lane basis.


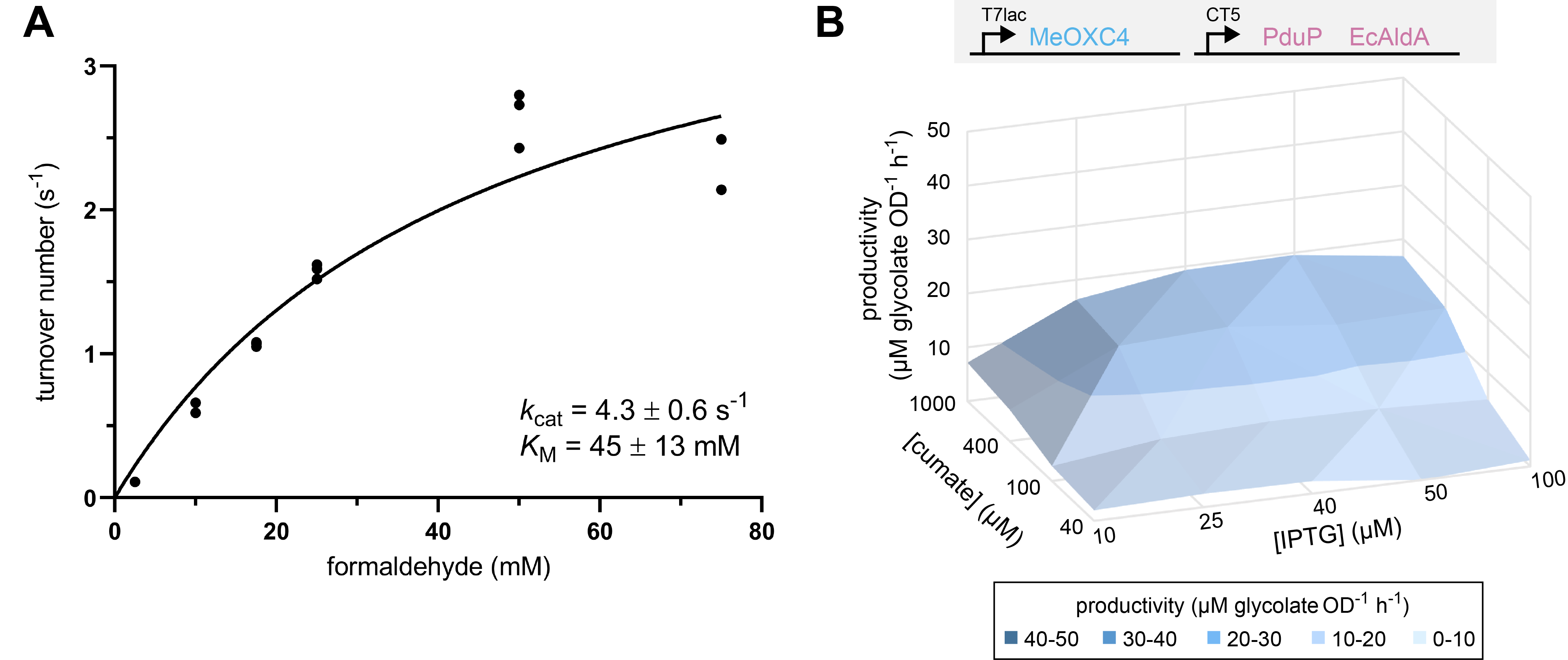


Figure S13. Effect of PduP as an ACR on the whole-cell conversion of formaldehyde to glycolate. A) Kinetic parameters for the conversion of formaldehyde to formyl-CoA. B) Glycolate production by *E. coli* whole cells expressing MeOXC4, PduP and LmACR under different inducer concentrations. Productivity and promoters are defined as in **Figure 6C**. Mean of n = 2 replicates is shown as a surface plotted against IPTG and cumate concentration.

# Supporting References

[1] Burgener, S., Cortina, N. S., and Erb, T. J. (2020) Oxalyl-CoA Decarboxylase Enables Nucleophilic One-Carbon Extension of Aldehydes to Chiral alpha-Hydroxy Acids, *Angew. Chem. Int. Ed. Engl.* *59*, 5526-5530.

[2] Trudeau, D. L., Edlich-Muth, C., Zarzycki, J., Scheffen, M., Goldsmith, M., Khersonsky, O., Avizemer, Z., Fleishman, S. J., Cotton, C. A. R., Erb, T. J., Tawfik, D. S., and Bar-Even, A. (2018) Design and in vitro realization of carbon-conserving photorespiration, *Proc. Natl. Acad. Sci. U.S.A.* *115*, E11455-E11464.

[3] Peter, D. M., Vogeli, B., Cortina, N. S., and Erb, T. J. (2016) A Chemo-Enzymatic Road Map to the Synthesis of CoA Esters, *Molecules* *21*, 517.

[4] Zarzycki, J., Sutter, M., Cortina, N. S., Erb, T. J., and Kerfeld, C. A. (2017) In Vitro Characterization and Concerted Function of Three Core Enzymes of a Glycyl Radical Enzyme - Associated Bacterial Microcompartment, *Sci. Rep.* *7*, 42757.

[5] Bradford, M. M. (1976) A rapid and sensitive method for the quantitation of microgram quantities of protein utilizing the principle of protein-dye binding, *Anal. Biochem.* *72*, 248-254.

[6] Kabsch, W. (2010) Integration, scaling, space-group assignment and post-refinement, *Acta Crystallogr. D.* *66*, 133-144.

[7] Winn, M. D., Ballard, C. C., Cowtan, K. D., Dodson, E. J., Emsley, P., Evans, P. R., Keegan, R. M., Krissinel, E. B., Leslie, A. G. W., McCoy, A., McNicholas, S. J., Murshudov, G. N., Pannu, N. S., Potterton, E. A., Powell, H. R., Read, R. J., Vagin, A., and Wilson, K. S. (2011) Overview of the CCP4 suite and current developments, *Acta Crystallogr. D.* *67*, 235-242.

[8] Adams, P. D., Afonine, P. V., Bunkoczi, G., Chen, V. B., Davis, I. W., Echols, N., Headd, J. J., Hung, L. W., Kapral, G. J., Grosse-Kunstleve, R. W., McCoy, A. J., Moriarty, N. W., Oeffner, R., Read, R. J., Richardson, D. C., Richardson, J. S., Terwilliger, T. C., and Zwart, P. H. (2010) PHENIX: a comprehensive Python-based system for macromolecular structure solution, *Acta Crystallogr. D.* *66*, 213-221.

[9] Emsley, P., and Cowtan, K. (2004) Coot: model-building tools for molecular graphics, *Acta Crystallogr. D.* *60*, 2126-2132.

[10] Kille, S., Acevedo-Rocha, C. G., Parra, L. P., Zhang, Z. G., Opperman, D. J., Reetz, M. T., and Acevedo, J. P. (2013) Reducing codon redundancy and screening effort of combinatorial protein libraries created by saturation mutagenesis, *ACS Synth. Biol.* *2*, 83-92.

[11] Neidhardt, F. C., Bloch, P. L., and Smith, D. F. (1974) Culture Medium for Enterobacteria, *J. Bacteriol.* *119*, 736-747.

[12] Chou, A., Clomburg, J. M., Qian, S., and Gonzalez, R. (2019) 2-Hydroxyacyl-CoA lyase catalyzes acyloin condensation for one-carbon bioconversion, *Nat. Chem. Biol.* *15*, 900-906.

[13] Kitagawa, M., Ara, T., Arifuzzaman, M., Ioka-Nakamichi, T., Inamoto, E., Toyonaga, H., and Mori, H. (2005) Complete set of ORF clones of Escherichia coli ASKA library (a complete set of E. coli K-12 ORF archive): unique resources for biological research, *DNA Res.* *12*, 291-299.

[14] Scheffen, M., Marchal, D. G., Beneyton, T., Schuller, S. K., Klose, M., Diehl, C., Lehmann, J., Pfister, P., Carrillo, M., He, H., Aslan, S., Cortina, N. S., Claus, P., Bollschweiler, D., Baret, J.-C., Schuller, J. M., Zarzycki, J., Bar-Even, A., and Erb, T. J. (2021) A new-to-nature carboxylation module to improve natural and synthetic CO2 fixation, *Nat. Catal.* *4*, 105-115.

[15] Berthold, C. L., Toyota, C. G., Moussatche, P., Wood, M. D., Leeper, F., Richards, N. G., and Lindqvist, Y. (2007) Crystallographic snapshots of oxalyl-CoA decarboxylase give insights into catalysis by nonoxidative ThDP-dependent decarboxylases, *Structure* *15*, 853-861.
